# Amplification through local critical behavior in the mammalian cochlea

**DOI:** 10.1073/pnas.2503389122

**Published:** 2025-07-14

**Authors:** Rodrigo G. Alonso, Francesco Gianoli, Brian Fabella, A. J. Hudspeth

**Affiliations:** ^a^HHMI, The Rockefeller University, New York, NY 10065; ^b^Laboratory of Sensory Neuroscience, The Rockefeller University, New York, NY 10065

**Keywords:** active process, hearing, hair cell, Hopf bifurcation, optical coherence tomography

## Abstract

Hearing hinges upon the ear’s ability to enhance its responsiveness by means of an energy-expending active process that amplifies the very mechanical inputs that it detects. This process is defined by four properties that, although seemingly unrelated, consistently occur together: amplification, sharp frequency tuning, compressive nonlinearity, and spontaneous otoacoustic emission. In nonmammal tetrapods, the active process is evident in individual hair cells. The hair bundles of the bullfrog, for example, exhibit all four attributes by operating near a Hopf bifurcation—a critical regime in which these properties naturally coalesce. In mammals, however, the delicate nature of the cochlea has restricted the evidence for an active process to studies in vivo, where it is generally attributed to the collective effort of the outer hair cells that energize the traveling wave along the cochlear spiral. As a result, the cellular mechanisms that underlie the properties of mammalian hearing remain contested, with uncertainty about whether criticality plays a role in the cochlea’s active process. Here we show that, when placed in a recording chamber that closely mimics the in vivo physiological environment, a segment of the mammalian cochlea ex vivo displays the features of the active process—amplification, frequency tuning, compressive nonlinearity, and the generation of distortion products. We show that this process operates locally, independently of traveling waves, and that the sensory epithelium achieves active amplification by operating near criticality at a Hopf bifurcation. The results reveal the existence of a unified biophysical principle that underlies auditory processing across species and even phyla.

## Significance

Uniquely among our sensory organs, the ear expends energy to amplify the very stimuli that it detects. This “active process” endows the cochlea with exceptional sensitivity, sharp frequency tuning, and broad dynamical range—yet its workings remain elusive. To date, the cochlea’s fragility and inaccessibility have confined studies in vivo, where global phenomena confound local dynamics. Bridging the gap between cellular and whole-organ behavior, we introduce a preparation that preserves the active process ex vivo in a cochlear segment and thus overcome a long-lasting experimental barrier. We show that the active process operates locally and that the sensory epithelium operates near criticality at a Hopf bifurcation. This result reveals a unified biophysical principle that underlies hearing across insects, nonmammalian vertebrates, and mammals alike.

The cochlea is a marvel of evolutionary engineering, a compact apparatus for resolving the spectra of sounds and encoding the information for processing by the brain. Central to cochlear function are hair cells, the extraordinarily sensitive sensory receptors of the internal ear (1, 2). Each hair cell is topped by a hair bundle, or cluster of mechanosensitive microvilli that pivot in response to sounds, the structure of which is remarkably conserved across vertebrates.

Like the cochlea as a whole (3–5), individual hair cells from nonmammalian tetrapods such as frogs exhibit four properties that together constitute the so-called active process (2, 5–7). First, the cochlea of a living animal displays mechanical responses a hundred to a thousand times as great as those in a dead animal, a feature that is thought to imply mechanical amplification (8). Individual hair cells ex vivo can demonstrably amplify mechanical inputs to their mechanoreceptive hair bundles (9, 10). A second feature of the cochlea is sharp frequency selectivity, which enables humans to discern frequency differences of 0.2% or less (11). Individual hair bundles are likewise tuned to specific frequencies of mechanical stimulation (12). Next, the cochlea displays compressive nonlinearity: In the cochlear region most sensitive to any specific frequency, the mechanical, electrical, and neural responses grow at a rate well below that of an increasing stimulus (13). Hair cells, too, display responses that scale as the cube root of the stimulus amplitude (14). Finally, in a quiet environment, 70% of normal human ears display spontaneous otoacoustic emission, the radiation of one or more pure tones from an unstimulated ear (15). Individual hair bundles also oscillate spontaneously at specific frequencies (16).

**Fig. 1.**
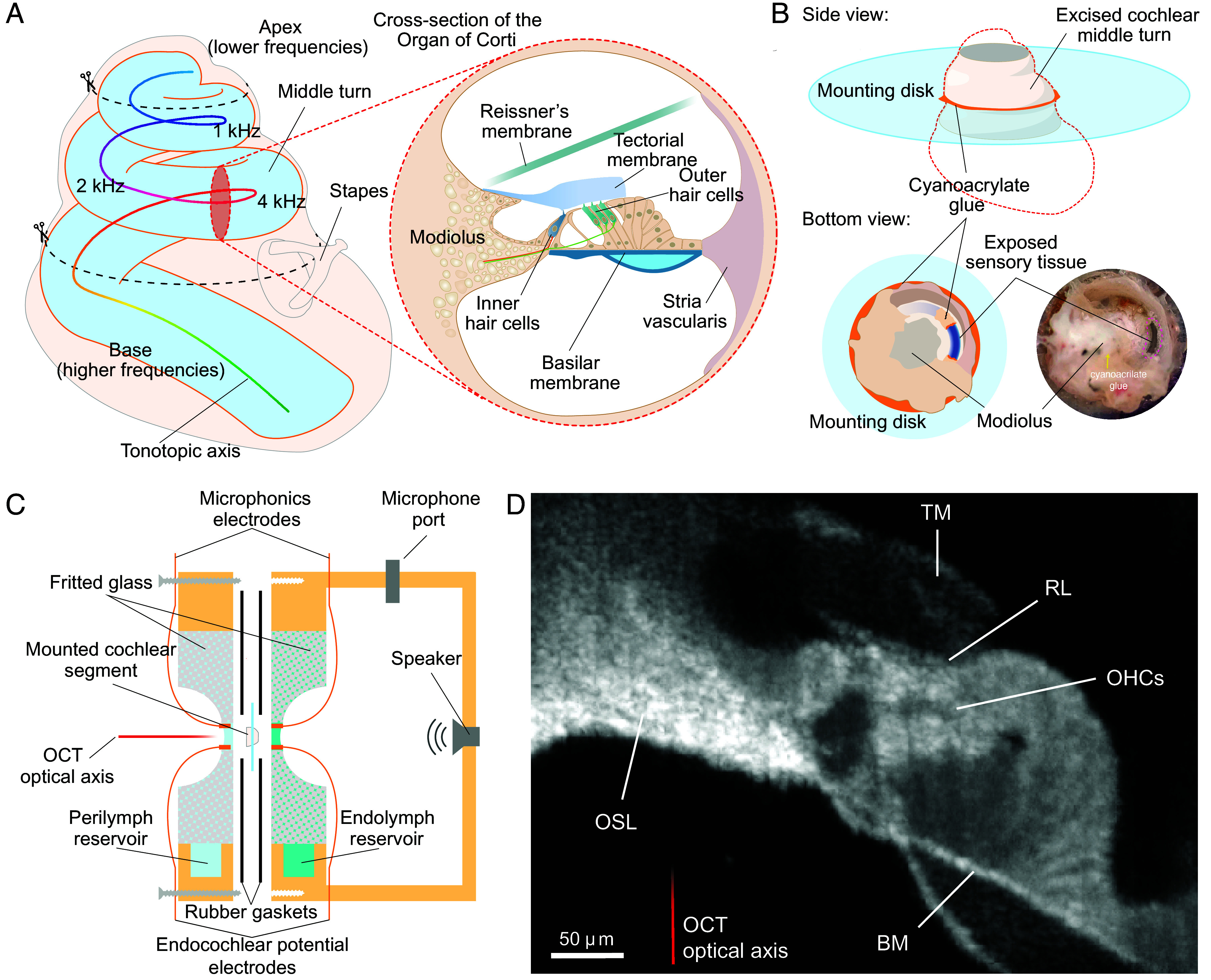
Excision and mounting of a cochlear segment. (*A*) A diagram of the tonotopic arrangement of the gerbil’s cochlea shows the range of frequencies represented in the middle turn. The two dashed black lines indicate where the cochlear bone was incised to isolate the turn. The *Inset*, a cross-section of the middle turn, details the arrangement of the organ of Corti and the location of the hair cells. (*B*) An excised middle turn, mounted on a plastic coverslip for experimentation, is shown schematically in side and bottom views, the latter of which is complemented by an image of the actual preparation. Cyanoacrylate adhesive (orange) secures the cochlear bone to the disk and seals the ends of the exposed cochlear segment. The modiolus is also coated in adhesive to separate the perilymph-bathed basolateral surface of the cochlear partition and the endolymph-bathed apical surface. To provide electrical and solute access to the scala media, Reissner’s membrane is torn open with a fine glass electrode. (*C*) A schematic diagram of the experimental apparatus details the recording chamber designed to simulate the normal cochlear environment. Each porous fritted glass disk with a hemispherical depression retains the endolymph or perilymph that it adsorbs from the associated reservoir. A central aperture in each disk accommodates the cochlear segment that protrudes from the mounting disk. A thin layer of liquid spans each side of the opening due to surface tension and bathes the segment. This arrangement ensures that the sensory tissue is exposed to consistent, small volumes of endolymph and perilymph while remaining connected to a larger reservoir for diffusional exchange of solutes. Silicone-rubber gaskets ensure a tight seal between the two compartments. A pair of pellet electrodes measures the microphonic response across the sensory tissue, and another pair maintains a constant potential difference across the partition to simulate the endocochlear potential. In the configuration shown, the perilymphatic side faces the OCT microscope, whereas the endolymphatic side faces a sealed chamber with a microphone and a speaker to deliver sound stimuli. (*D*) A static optical cross-section (B-scan) of the cochlear partition, captured by OCT, shows the organ of Corti ex vivo. Largely transparent to the light beam, the tectorial membrane (TM) rests atop the reticular lamina (RL), from which the hair bundles extend. The highly reflective somata of OHCs border the darker Deiters’ cells below. The pectinate zone of the basilar membrane (BM) extends from the osseous spiral lamina (OSL) and includes a broad region filled with dark material, a characteristic of gerbilline rodents.

In the mammalian cochlea, the active process is attributed to the working of the outer hair cells (OHCs), which reside in the Organ of Corti alongside inner hair cells (Fig. 1). Whereas inner hair cells are the primary sensory transducers, responsible for nearly all afferent signaling to the brain, OHCs can elongate and contract their somata in response to transmembrane voltage changes through a process called electromotility—a capability demonstrated in isolated cells (17). OHCs participate in a feedback loop: Sound-induced vibrations in the cochlea deflect the cell’s hair bundle and modulate the opening of mechanosensitive ion channels, current through which alters the cell’s membrane potential (18, 19). This voltage change drives an electromotile response that amplifies the vibration of the organ of Corti on a cycle-by-cycle basis. Because electromotility is largely a linear process (18), the compressive nonlinearity arises because of the sigmoidal relationship between hair-bundle displacement and the mechanotransduction currents (18, 20): Small displacements elicit a linear electromotive response, but as deflection increases, channel gating saturates and the response grows sublinearly (18, 19). Precise phase timing is essential for this mechanism to effectively amplify vibrations. It remains unclear how electromotility could ensure such accuracy at the frequencies of mammalian hearing (21, 22).

In a nonmammalian hair cell, the seemingly disparate features of the active process are achieved by means of a well-understood feedback mechanism that keeps the hair bundle on the verge of a mechanical instability termed the Hopf bifurcation (23–26). The four cardinal features of the active process arise naturally and represent distinct facets of the same underlying mechanism. When a dynamical system is poised at the boundary between two distinct dynamical regimes, it can exhibit critical behaviors that do not occur in either of those regimes. At a Hopf bifurcation, for example, the system’s sensitivity is strongly enhanced. Frequency selectivity can greatly exceed that for the bulk phases. The scaling of responses can be highly nonlinear, with outputs growing as the cube root of the inputs. Critical behavior appears to be common in biological systems (27–30) and offers clear behavioral advantages (28), especially in sensory systems such as those involved in hearing (31).

Although the cochlea as a whole displays all the hallmarks of the active process (3, 8), the relationship between the behavior of individual hair cells and the macroscopic auditory properties remains uncertain (21, 32). It is also unclear whether the principle of criticality observed in nonmammalian species—that of the Hopf bifurcation (8)—also underpins the active process in the mammalian cochlea.

Even the existence of active amplification within the cochlea remains a topic of contention. The compressive nonlinearity observed in vivo, for example, can be interpreted as the result of cumulative dampening as traveling waves propagate along the cochlear spiral (21, 33) rather than as evidence for a biological process that amplifies vibrations that contribute net mechanical work (21, 33).

There are two principal reasons for this impasse. First, because of the technical difficulties associated with research on the relatively inaccessible and extremely delicate mammalian cochlea, the active process of individual hair cells has largely been investigated in the simpler ears of nonmammalian vertebrates such as frogs and turtles. Second, the active process of the mammalian cochlea is complicated by traveling waves (5). Acoustic stimulation at a specific frequency does not affect only a limited number of hair cells; instead, the sound engenders a mechanical wave that originates at the cochlear base and grows as it propagates toward the apex. The wave diminishes in wavelength and increases in amplitude as it approaches a frequency-specific place, where it collapses by transferring much of the acoustic energy to hair cells. Moreover, the wave’s propagation along the cochlear spiral involves complex motions within the organ of Corti (34) and the overlying tectorial membrane (TM) (35, 36). It follows that the cochlear active process is not a localized phenomenon but that numerous hair cells along the path of the traveling wave shape its form and magnitude (37).

In this work, we examine the mechanical and electrical activity of isolated segments of a mammalian cochlea ex vivo under conditions that preserve key physiological features and closely replicate the in vivo environment. In the absence of a traveling wave, yet with both inner and OHCs operating within the normal anatomical and physiological context of the organ of Corti, we seek to isolate the local cellular mechanisms that underscore cochlear dynamics. More broadly, this approach allows us to ask whether the principle of criticality also underlies mammalian hearing and to explore the common behaviors that emerge from operation in a critical dynamical regime.

## Results

To investigate the active process of the mammalian cochlea ex vivo, we isolated cochlear segments 500 μm to 1,000 μm in length from the gerbil (*Meriones unguiculatus*), a species that exhibits significant overlap in hearing range with humans (38) (*SI Appendix*, Fig. S1). Each segment was mounted in a two-compartment experimental chamber that replicated the physiological environment of the intact cochlea (Fig. 1 *B* and *C* and *SI Appendix,* Fig. S2). In one compartment endolymph bathed the apical epithelial surfaces, whereas in the other compartment perilymph contacted the basolateral cellular surfaces. We warmed the preparation to the normal body temperature (39) of 38 ˚C and provided a steady endocochlear potential (40) of 80 mV to 100 mV, with the endolymphatic compartment positive with respect to the perilymphatic one. Hair cells were stimulated by sound pressure in an air-filled compartment separated from the external atmosphere by the cochlear segment (Fig. 1*C* and *SI Appendix*, Fig. S2). We measured sound pressure and the summated electrical response of the hair cells, known as the cochlear microphonic potential (41), building on our previous work (42), as well as the mechanical vibrations of the cochlear partition.

A key simplification afforded by our system was the suppression of traveling waves. In the intact cochlea, sound-induced vibrations propagate as traveling waves along the 11.5 mm spiral of the gerbil’s organ of Corti (43). In an isolated segment, we ordinarily exposed only 4 to 8% of the total length (Fig. 1*B*).

By removing both the bony ceiling and floor of the second turn, we gained unobstructed optical access to the organ of Corti, enabling us to visualize active tissue with resolutions unattainable in vivo (Fig. 1*D* and *SI Appendix*, Fig. S3). High-speed video micrography (44), theoretical analysis (45), and OCT vibrational measurements (*SI Appendix*, Fig. S4) independently demonstrate that a cochlear segment oscillates as a single unit and exhibits minimal phase differences along its length. This experimental configuration enabled us to study the active process in the absence of traveling waves.

A mathematical analysis of the Hopf bifurcation provides four well-defined predictions to test experimentally whether a cochlear segment is poised near criticality. First, the response of a dynamical system near such a bifurcation grows with a 1/3 slope on a doubly logarithmic scale as a function of the stimulus force when driven at its characteristic frequency (CF). Second, this power-law behavior is restricted to stimulus frequencies near the local CF. The relationship becomes linear for frequencies substantially greater or less than the CF. A system operating near a Hopf bifurcation thus unequivocally differs from one with a saturating nonlinearity, for in the latter instance, the slope does not depend on the stimulus frequency. Third, a critical system’s sensitivity peaks on the brink of a Hopf bifurcation. Detuning the cochlear segment, for example by altering physiological conditions, should decrease and linearize the response. Finally, a critical system displays cubic distortion products when stimulated simultaneously by two primary tones with nearby characteristic frequencies (6). Furthermore, these distortion products exhibit sublinear growth as the stimulus level increases: They maintain a consistent magnitude of 10 to 20% relative to the responses for the primary tones (46).

### Amplification, Frequency Tuning, and Compressive Nonlinearity.

To assess frequency tuning in a cochlear segment, we presented zwuis multitone stimuli that permitted the simultaneous characterization of responses at several frequencies (47, 48). This strategy prevented misrepresentation of the contribution of individual tones in tuning curves by ensuring that no primary tone stimulus overlapped with a combination tone (distortion product) that was generated by the system’s nonlinearity when the primary tones interacted.

**Fig. 2.**
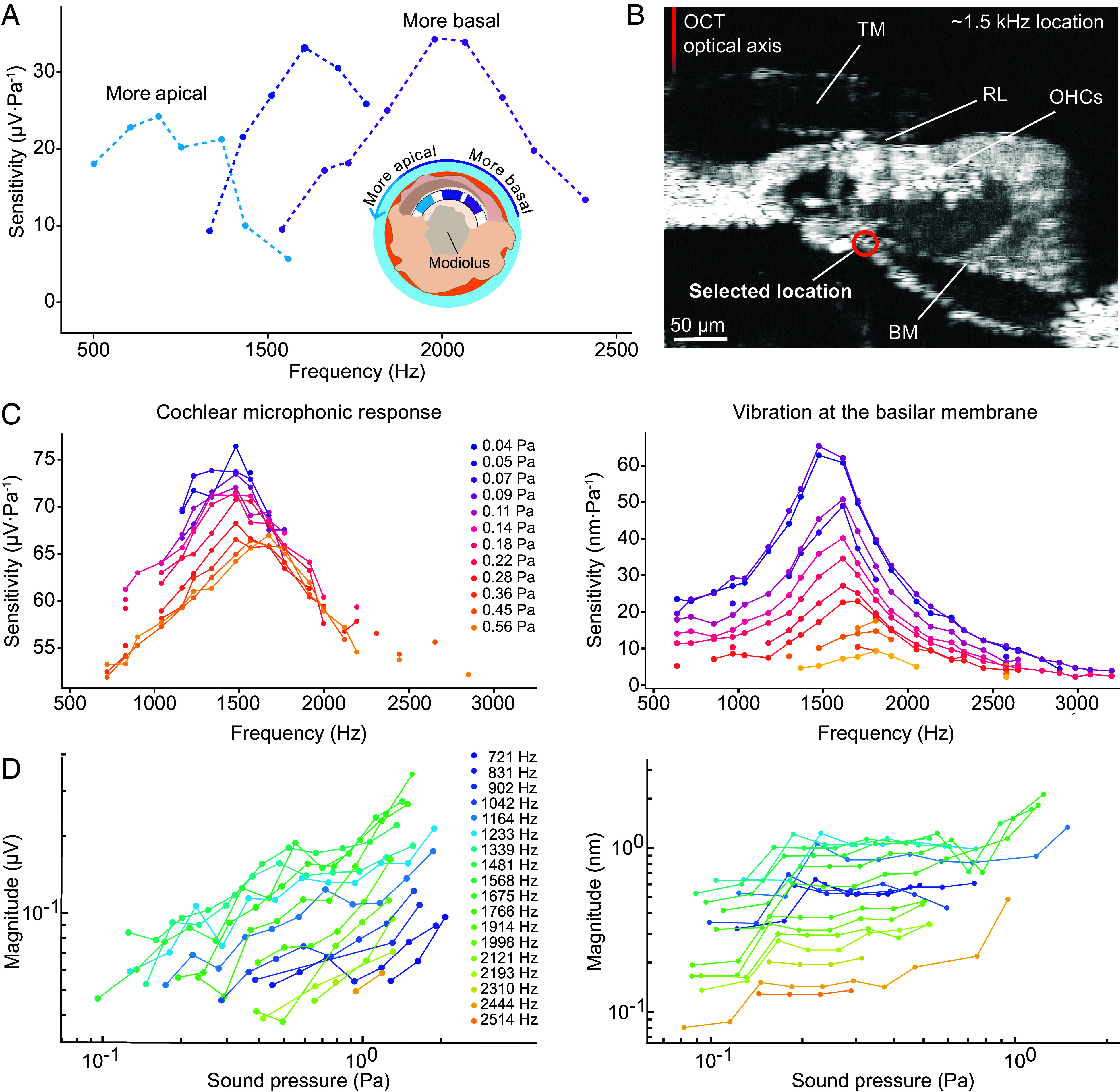
Frequency tuning and compressive amplification in a cochlear segment. (*A*) The sensitivity of the electrical response–the microphonic potential divided by the magnitude of the stimulus pressure–is shown as a function of frequency in three representative cochlear segments corresponding to different locations along the tonotopic map. The sensitivities of the segments peak at successively greater frequencies at progressively more basal positions. (*B*) An OCT image depicts a radial slice of the organ of Corti at the center of a cochlear segment near the 1.5 kHz tonotopic location. Relevant anatomical traits are indicated as shown in Fig. 1*D*. The red circle marks the specific position at which vibration profiles parallel to the OCT optical axis were acquired. (*C*) For cochlear microphonic (*Left*) and vibratory (*Right*) responses to multifrequency stimuli of different amplitudes, the sensitivity peaks at a CF near 1,500 Hz. The curves at different stimulus levels separate near the CF, for which the growth is nonlinear. As expected for a dynamical system near criticality, however, the curves converge at significantly greater or lesser frequencies. (*D*) On doubly logarithmic axes and as functions of the magnitude of stimulation, both the cochlear microphonic response (*Left*) and the vibration of the cochlear partition (*Right*) display sublinear logarithmic slopes that reach one third at the CF. Away from that frequency, the slopes progressively approach unity, which signifies linear responsiveness.

With these stimuli, cochlear microphonic responses and vibrational measurements of the organ of Corti demonstrated frequency-tuned behavior (Fig. 2 and *SI Appendix*, Fig. S5). Consistent with the tonotopic map (43), apical segments were tuned to lower frequencies, whereas basal segments responded to higher frequencies (Fig. 2*A*). The sensitivity of each preparation was quantified as the ratio of the cochlear microphonic response or basilar membrane (BM) motion to the magnitude of stimulation (Fig. 2 *C* and *D*). Direct comparison of the measured sound‐pressure levels with those conventionally measured in vivo is precluded by the configuration of our system. In particular, the removal of the middle ear and the abolition of the traveling wave are estimated to reduce the effective stimulus amplitude by at least 26 dB relative to stimulation with the same sound pressure in vivo (*SI Appendix, *Extended Materials and Methods**).

Sensitivity curves exhibited striking nonlinearity: Low-intensity stimuli elicited proportionately larger responses than high-intensity stimuli, a result consistent with the cochlea’s compressive dynamics. Near the CF of each segment, responses grew sublinearly, reflecting the cochlea’s active amplification process (Fig. 2 *C* and *D*). In contrast, responses to more distant frequencies displayed more linear behavior consistent with passive mechanical properties. These findings accord with in vivo data on BM motion and neural activity in mammals, for which electrical responses mirror mechanical responses (49).

Notice that when a Hopf oscillator is driven by a zwuis stimulus, the frequency components compete and interact through the oscillator’s nonlinearity and thus produce a stronger compression than would single-tone stimulation. As the number of simultaneous tones increases, the growth of response amplitude progressively flattens and approaches level-independence in the white-noise limit (50). These intermodulation effects obscure the oscillator’s intrinsic nonlinear signature, and complicate the interpretation of its dynamic properties. When stimulating a cochlear segment with zwuis stimuli, we consistently observed sublinear growth with a lower slope compared to single-tone stimulation at the same frequency (Fig. 2*D*). For this reason, we employed zwuis stimuli only to initially assess the CF of a segment.

### Power-Law Scaling of the Cochlear Microphonic Potential.

**Fig. 3.**
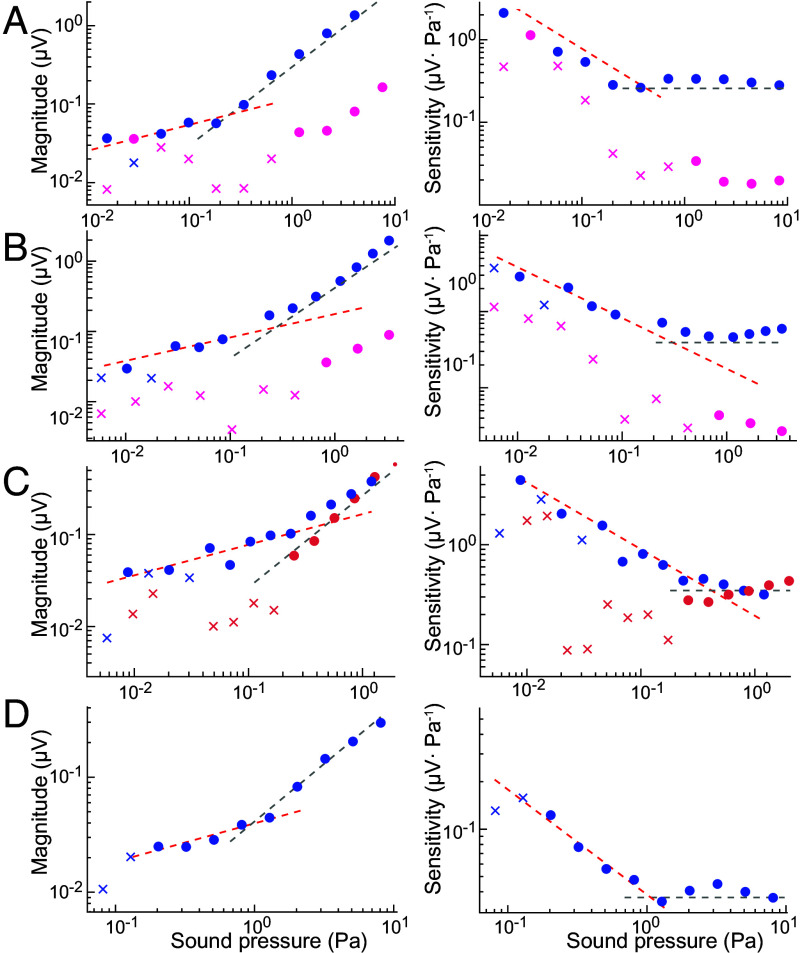
Power-law behavior in microphonic responses. (*Left*) Examples of microphonic potential magnitudes in response to a single-tone stimulus at the CF are shown as a function of sound pressure level for four different preparations (blue circles). The responses show sublinear growth, with a logarithmic slope close to 1/3 (red dashed line), followed by linear growth at high sound intensity (gray dashed line). (*Right*) The same data are displayed as sensitivities as a function of sound-pressure level. In this case, the sublinear growth indicated by the red dashed line has a slope of −2/3, and the linear increase is represented by the gray dashed line with a null slope. The dots represent phase-locked responses that pass a Rayleigh test (*P* < 0.001), whereas crosses indicate responses that are statistically indistinguishable from noise (*p* > 0.001). (*A*) (CF = 1,800 Hz, EP = 100 mV, blue circles) and (*B*) (CF = 1,040 Hz, EP = 100 mV; blue circles), When the endocochlear potential is absent (EP = 0 mV; pink circles), the responses fall into the noise but emerge linearly at high sound intensities. (*C*) (CF = 955 Hz, EP = 100 mV; blue circles), During stimulation at frequencies that are far from the CF (off CF = 2055 Hz, bright red circles), the response grows linearly. (*D*) A control preparation is shown for comparison (CF = 1,840 Hz, EP = 100 mV).

**Fig. 4.**
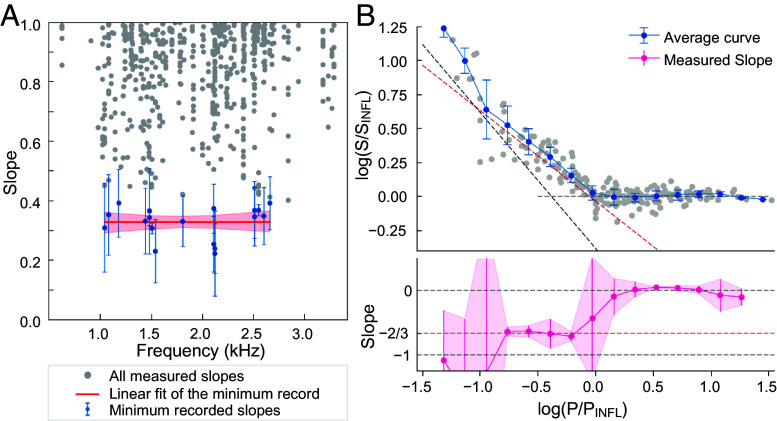
Power-law behavior in microphonic responses across cochlear segments. (*A*) All slope measurements extracted from the microphonic responses of cochlear segments in 78 experiments (48 gerbils), plotted as a function of stimulus frequency. For each experiment, the microphonic response (response amplitude versus sound pressure level) was analyzed using a sliding window across the stimulus–intensity axis, yielding multiple local slope estimates per response curve. These slopes were computed by fitting consecutive and overlapping triplets and quadruplets of adjacent data points in doubly logarithmic coordinates. As a result, each experiment contributed many slope values—all of which are reported (gray dots). The minimum slope per experiment was extracted using a moving window of 500 Hz with a step size of 10 Hz; Hz (blue dots with error bars) as an objective measure of maximal compression. Under a null model of saturating responses, slope values would scatter without a consistent lower bound; repeated minima near 1/3 indicate instead a power-law scaling characteristic of a system near a critical point. The frequencies explored went from approximately 600 Hz to 3,300 Hz. All slopes whose SE was larger than 0.15 were excluded from the analysis. The smallest recorded values were encountered in a frequency range of 1 to 2.5 kHz, congruent with the location of the exposed cochlear segments along the tonotopic axis. The linear fit on the slopes (red line, with 95% CI in pink) is y = mx + q, with m = 0.0 ± 0.0, and q = 0.32 ± 0.03. The mean of the smallest slopes recorded across experiments was 0.328 ± 0.009 with a 95% CI of 0.31 to 0.35. (*B*, *Top*) Gray dots show the sensitivity S of the microphonic response (FFT magnitude/sound pressure) as a function of sound pressure *P* (in doubly logarithmic coordinates) for multiple cochlear segments that exhibited a sublinear response (any slope < 0.5). Each dataset was fit with a piecewise linear model—one segment having a negative slope, the other a null slope—and aligned based on their inflection points (P_INFL_,S_INFL_). The blue circles represent the mean of the aligned curves, and error bars indicate the corresponding SD. (*B*, *Bottom*) Pink circles show the local slope of the average curve in the *Top* panel, calculated by fitting the average data points within a four-point sliding window. The vertical error bars show the half-width of the 95% CI estimated by a *t* test (*SI Appendix*, Extended Materials and Methods**). The black dashed lines follow a slope −1, red dashed lines a slope of −2/3—that expected by a dynamical system near a Hopf bifurcation—while gray dashed lines mark a null slope.

To investigate the compressive behavior of isolated cochlear segments, we examined their microphonic responses to single tones of varying intensity. Our experiments revealed that, at the CF, the microphonic response exhibited a pronounced compressive nonlinearity. When plotted on doubly logarithmic coordinates, the response grew sublinearly with a slope of approximately 1/3 (Fig. 3 and *SI Appendix,* Fig. S6). This behavior echoed in vivo observations of basilar-membrane mechanics (51) and the responses of individual hair cells in nonmammalian vertebrates (14). This power law pertained to stimuli near the CF at which the cochlear segment was tuned, whereas stimulation at other frequencies yielded progressively more linear responses with slopes closer to unity (Fig. 3 and *SI Appendix*, Fig. S6). The corresponding sensitivity relationships scaled as the negative two-thirds power of the stimulus amplitudes (Figs. 3 and 4 and *SI Appendix*, Fig. S6).

Altering a preparation’s environment, for example by eliminating the endocochlear potential, yielded linear responses (Fig. 3 and *SI Appendix*, Fig. S6). Moreover, stimulation without an endocochlear potential yielded reduced amplitude vibrations, indicative of passive responses (*SI Appendix*, Fig. S7). We also observed multiple instances of power-law slopes greater than one-third; these steeper slopes frequently appeared in inadequately dissected samples (*SI Appendix*, Fig. S8 *A*–*D*) and in initially healthy preparations as the experiment progressed, particularly following significant stimulation of the hair cells (*SI Appendix*, Fig. S8*E*). In both instances, the sensory tissue showed visible signs of damage. Such progressive linearization of the responses mirrored what has been observed in vivo (51–53).

In 78 experiments on 48 gerbils, we stimulated cochlear segments with pure tones at frequencies ranging from approximately 600 Hz to 3,000 Hz. To obtain an unbiased estimate of the maximum compressive growth of the cochlear microphonic responses, we grouped data points in consecutive and overlapping triplets and quadruplets and performed a linear fit in doubly logarithmic coordinates. Fits were retained only if the SE of the slope did not exceed 0.15. This procedure yielded a distribution of slopes across the entire frequency range explored (Fig. 4*A* and *SI Appendix*, Fig. S9). Due to the unbiased search, each experiment contributed many slope values, all of which we report (Fig. 4*A*). Because cochlear microphonic responses in most experiments extended well into the linear regime at higher sound pressure levels, slopes close to unity are overrepresented in this analysis. However, the mean lower-bound slope observed was 0.33 ± 0.10 (Fig. 4*A* and *SI Appendix*, Fig. S9 and *Extended Materials and Methods*), and it was limited to stimulus frequencies between 1 kHz and 2.5 kHz—slightly below the range of 1 to 4 kHz range typical of the gerbil’s middle turn (43) but consistent with the portion of the tonotopic axis accessible in our experiments. This approach is conservative: Under a null model in which responses arise either from a saturating nonlinearity or from a linear response that gradually emerges from noise, local slope values would vary without a consistent lower bound. Instead, the distinct clustering of minimum slopes around 1/3 strongly supports a Hopf-type compressive nonlinearity.

Comparing absolute response magnitudes across different preparations is challenging due to variations among animals, differences in cochlear dimensions, and other factors. To obtain an average response, we focused on those responses that exhibited any nonlinear behavior (defined as any slope smaller than 0.5). For each such curve, we plotted the sensitivity S as a function of the sound-pressure level *P* and used an unsupervised algorithm to identify an “inflection point” (P_INFL_, S_INFL_) separating the two regimes (*SI Appendix, *Extended Materials and Methods**). Each sensitivity curve was then shifted in the log–log space so that their inflection points aligned. To determine the average trend across experiments, we applied a running average along the frequency axis to obtain the average sensitivity curve (Fig. 4 *B*, *Top*). The average slope was calculated by fitting data points within a sliding window (*SI Appendix*, Fig. S9). This analysis reveals a nonlinear region in the sensitivity with a characteristic slope close to −2/3.

The fact that cochlear segments displayed increased sensitivity and a relationship of response magnitude to stimulus size that was strikingly nonlinear over a range of weak to moderate stimulation intensities is consistent with the operation of an active process in the mammalian cochlea. Because traveling waves were absent in our preparation (*SI Appendix,* Fig. S4), the observed amplification could not be interpreted as the accumulation of dampening along the cochlear spiral.

### Distortion Products.

Distortion-product otoacoustic emissions, also called combination tones, are generated by the inner ear in response to a pair of simultaneously presented pure tones. When sounds are played at a higher primary frequency *f*_2_ and a lower one *f*_1_, an individual can readily hear the cubic combination tones 2·*f*_1_ − *f*_2_, 2·*f*_2_ − *f*_1_, 2·*f*_1_ + *f*_2_, and 2·*f*_2_ + *f*_1_ as well as higher-order tones that are progressively weaker. These frequencies, which are generated by nonlinearities within the cochlea and appear in basilar-membrane movements (54, 55), are an intrinsic feature of our hearing. The first to notice them was the violinist Giuseppe Tartini in 1754 (56). Playing two notes while tuning his instrument, he realized he could hear a third note that he was not sounding. Contemporary physicians measure these emissions to evaluate cochlear function and assess hearing deficits in infants.

**Fig. 5.**
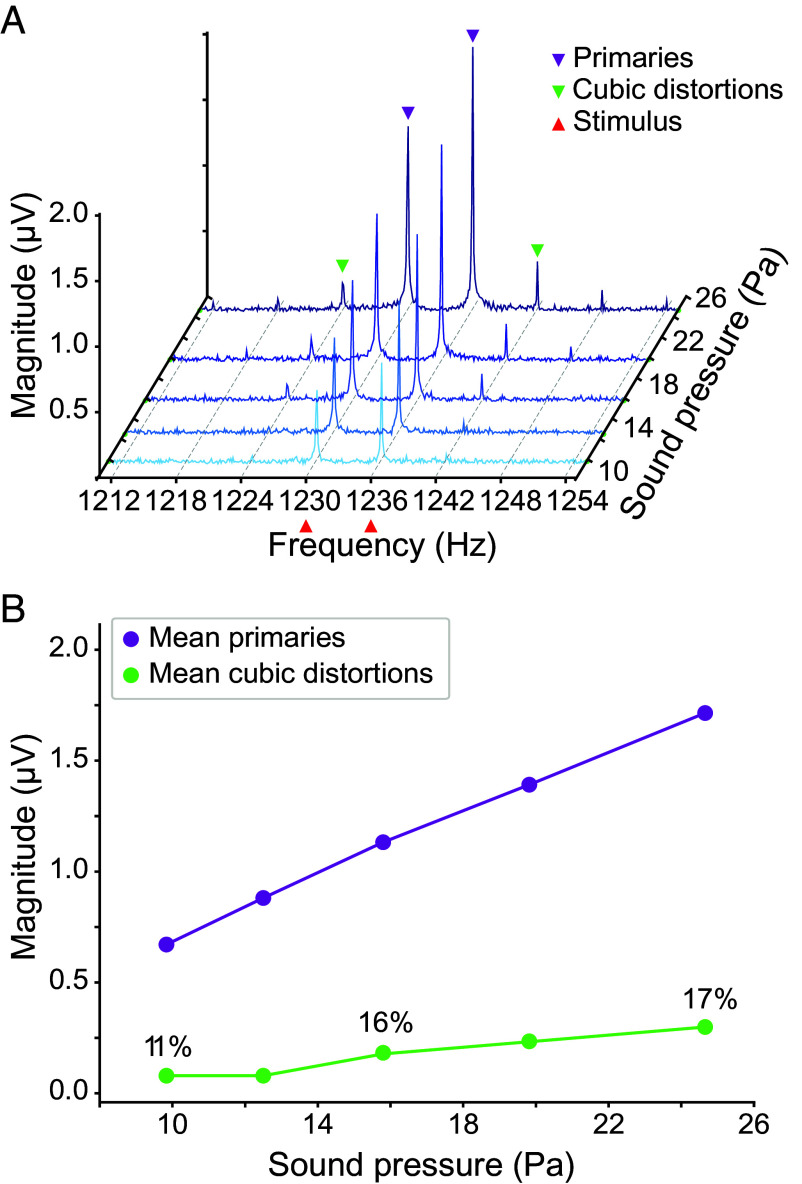
Cubic distortion products from a cochlear segment ex vivo. (*A*) Starting from the bottom, five plots portray the Fourier transforms of the cochlear microphonic responses for progressively increasing levels of stimulation. The primary tones were delivered through independent speakers at *f*_1_ = 1,230 Hz and *f*_2_ = 1,236 Hz (red arrowheads). Microphonic responses to the primary tones are evident from the outset (purple arrowheads). The first distortion products to emerge from the noise occurred at 2·*f*_1_ − *f*_2_ and 2·*f*_2_ − *f*_1_ (green arrowheads). Responses at 3·*f*_1_ − 2·*f*_2_ and 3·*f*_2_ − 2·*f*_1_ appeared at higher levels of stimulation. (*B*) Progressively stronger stimuli increased the responses both at the primary frequencies (purple) and those of the cubic distortion products (green). The relative growth—the mean responses of the distortion products divided by those of the primary tones—averaged 15 ± 3% and was relatively independent of the stimulus level.

By simultaneously delivering two tones into the experimental chamber through independent speakers, we elicited distortion products from cochlear segments ex vivo. We chose frequencies near a segment’s CF and used power spectra to monitor cochlear-microphonic responses at the primary frequencies and over a range of nearby frequencies. The first distortion products to emerge with a growing intensity of stimulation were 2·*f*_1_ – *f*_2_ and 2·*f*_2_ – *f*_1_, whose magnitudes were about 15% of those of the primary tones (Fig. 5). As the stimulation intensity increased, the distortion products rose at a consistent ratio to the primary responses. In separate experimental trials in which we evaluated distortion product generation at different sound pressure intensities, we observed cubic combination tones whose magnitudes averaged 12.8 ± 7.8% relative to the primary tones (mean ± SD, four experimental preparations; see *SI Appendix,* Table S1). This behavior resembles that observed in individual hair cells from the bullfrog (46). Moreover, higher-order distortion products were observed at increased stimulation levels (Fig. 5).

### Nonlinear Growth of Motion in the Organ of Corti.

The nonlinear characteristics of the cochlear segment and its microphonic responses indicate that the micromechanics of the organ of Corti likely exhibits nonlinear traits as well. By utilizing OCT, we could analyze the vibration patterns of specific cellular elements and establish a direct connection between their mechanical functions and electrical responses.

**Fig. 6.**
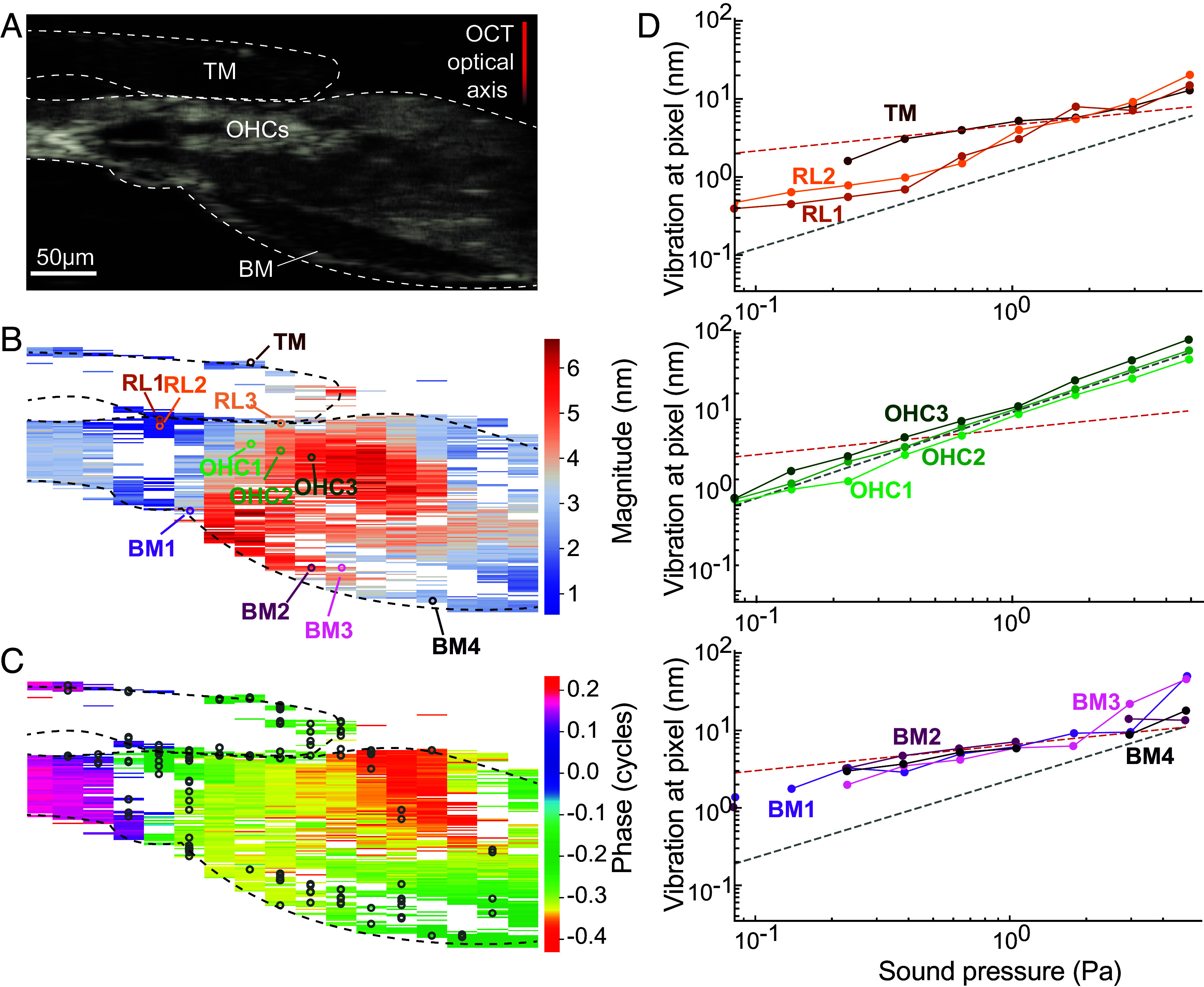
Nonlinear vibration within a cochlear segment. (*A*) In an OCT image of a radial section centered within a cochlear segment, the boundaries of the organ of Corti, BM, and TM are marked by a dashed white line. The TM, BM, and OHCs are labeled for reference. (*B*) The tuned vibration of the cochlear segment at the 2,410 Hz CF measured at a pressure of 0.39 Pa. Only pixels with statistically significant responses are shown. The radial slice was resolved by moving the scanning beam across twenty equally spaced points and by repeating the procedure for each 1 s pure-tone stimulus at the CF. The magnitudes are logarithmically scaled and presented as a heatmap. Colored circles represent particular pixels whose level-dependent vibrations are depicted in panel *D*. (*C*) In a phase map of the vibration response of the same radial section, black circles mark the pixels that—when their vibration amplitude is plotted as a function of the sound-pressure level—display at least three consecutive points with sublinear growth. (*D*) To illustrate the micromechanical complexity of the cochlear segment, vibration magnitude is plotted as a function of sound pressure for selected pixels located in three distinct positions: the TM and RL (*Top*); the region of OHC (*Middle*) and the BM (*Bottom*).

During sinusoidal stimulation of the cochlear segment, we observed nanometer-scale vibrations in various parts of the organ of Corti including the reticular lamina (RL) and TM (Fig. 6 and *SI Appendix*, Fig. S10). The high resolution of our system allowed us to pinpoint areas where vibrations at the CF showed sublinear growth that accorded with the compressive nonlinearity of the microphonic response. The regions that exhibited sublinear behavior were located primarily at the boundary between the TM and RL and at the BM (Fig. 6). Furthermore, regions that displayed increased movement occurred between the OHCs and Deiters’ cells (Fig. 6 and *SI Appendix*, Figs. S7, S10, and S12). These mechanical “hotspots,” characterized by elevated vibration within the region of OHCs, accord with in vivo observations (34), a correspondence that affirms the integrity of our preparation and emphasizes the contribution of OHCs to cochlear mechanics.

### The Hopf Bifurcation.

Our experiments revealed that isolated segments of the gerbil’s cochlea ex vivo exhibit three hallmarks of the active process: amplification, frequency tuning, and compressive nonlinearity (Figs. 2–5). Although distinct in their manifestations, these emergent properties can be cohesively interpreted within the framework of a Hopf bifurcation. This mathematical construct describes a dynamical system poised near a critical point at which the behavior of the system changes drastically—from quiescence to self-sustained oscillation—in response to small changes in a parameter of the system. Near the bifurcation point—at criticality—the system’s behavior can be fully described by the Hopf normal form

[1] $$\dot{z}\left(t\right)=\left(\epsilon +i\omega_{c}\right)z-\beta {\left|z\right|}^{2}z.$$

Here *z* is a complex variable representing the system’s state, *ε* is a real parameter controlling the system’s proximity to the bifurcation, $\omega_{c}$ is the natural frequency of oscillation, and *β* is a positive complex constant that controls the nonlinear term.

The Hopf normal form can be decomposed into three components: exponential growth or decline ($\dot{z}\left(t\right)=\epsilon z$), oscillation ($\dot{z}\left(t\right)=i\omega_{c}z$), and a cubic term ($-\beta {\left|z\right|}^{2}z$). The first two terms describe an oscillation whose amplitude grows exponentially for ε > 0, or decays exponentially to zero for ε < 0. The negative cubic term prevents these oscillations from growing indefinitely in amplitude. When ϵ<0, the system’s equilibrium is stable, and perturbations decay over time. As *ε* increases and exceeds zero, the equilibrium becomes unstable and the system begins to oscillate spontaneously at the natural frequency $\omega_{c}$. Close to the bifurcation point $\left(\epsilon ≲0\right)$, the dynamical system operates in a critical state at which its response displays the three cardinal features of the active process: amplification, frequency tuning, and compressive nonlinearity with a one-third power law. In the context of hearing, this regime elucidates the cochlea’s response to sinusoidal stimuli. If a pure-tone stimulus exerts a sinusoidal force $F\left(t\right)=F_{0}e^{+i\omega t}$,

[2] $$\dot{z}\left(t\right)=\left(\epsilon +i\omega_{c}\right)z-\beta {\left|z\right|}^{2}z+F_{0}e^{+i\omega t}.$$

We seek a steady-state solution of the form $z\approx Ae^{+i\omega t}$, which yields $\left|A\right|\sqrt{{\left(\omega_{c}-\omega \right)}^{2}+{\left|A\right|}^{4}}=\left|F_{0}\right|$. If the stimulus frequency is close to the natural frequency $\left(\omega =\omega_{c}\right)$, the magnitude of the response is expected to grow sublinearly as a function of the stimulus intensity. $\left|A\right|={\left|F_{0}\right|}^{1/3}$ The sensitivity should accordingly decline as $S=\left|A\right|/\left|F_{0}\right|={\left|F_{0}\right|}^{-2/3}$. As observed for cochlear segments, the responses should become linear away from the CF: $\left|A\right|=\left|F_{0}\right|/\left|\omega_{c}-\omega \right|$ for $\left|\omega_{c}-\omega \right|\gg 1$.

The Hopf framework also accounts for the generation of distortion products. These arise when primary tones of frequencies *f*_1_ and *f*_2_ are delivered simultaneously and reveal the most pronounced responses at the frequencies 2·*f*_1_ – *f*_2_ and 2·*f*_2_ – *f*_1_. When a dynamical system described by Eq. **1** is driven by two sinusoidal forces ${F\left(t\right)=F}_{1}e^{+i\omega_{1}t}{+F}_{2}e^{+i\omega_{2}t}$, in which $\omega_{1}=2\pi f_{1}$ and $\omega_{2}=2\pi f_{2}$, the nonlinear cubic term produces intermodulation terms at frequencies $2f_{1}-f_{2}$ and $2f_{1}-f_{2}$ and at higher orders. In proximity of the bifurcation $\left(\epsilon ≲0\right)$, the magnitude of these distortion products is predicted to grow at an approximately fixed ratio relative to the primary tones, as we observed in cochlear segments (Fig. 5). Notice that other nonlinearities, such as the saturations caused by the sigmoid activation curve of hair-cell mechanotransduction channels—central to the electromotile model of cochlear amplification—can also cause cubic distortions. However, these saturation-based distortions are expected to grow superlinearly with respect to the primary tones as the stimulation level rises (46), in contrast to the sublinear scaling predicted by models based on a Hopf bifurcation.

## Discussion

The cochlea displays remarkable features for any physical system, let alone a biological one. These properties define the so-called active process, whose cellular mechanisms in mammals have remained elusive, largely owing to the fragility, anatomical inaccessibility, and complexity of the cochlea, which have precluded direct experimental access.

Although challenging in its execution, the approach that we adopted to overcome these obstacles is conceptually straightforward: to isolate a functional segment of the organ of Corti as an ex vivo preparation devoid of traveling waves and to investigate the local dynamics underlying cochlear amplification. In this reduced ex vivo system, we observed key features of the active process: amplification, frequency tuning, compressive nonlinearity, and distortion products. These arose within a strip of sensory epithelium about 500 µm long, demonstrating that the active process does not necessitate traveling waves but can manifest locally. In this context, the nonlinear gain we observed cannot be explained as cumulative dampening or energy densification but points instead to the existence of a local process of amplification within the mammalian sensory epithelium.

Isolated segments of the gerbil’s cochlea revealed three manifestations of an active process, consistent with the dynamics of a dynamical system operating near a Hopf bifurcation—a critical regime in which enhanced responses to stimuli, frequency tuning, and nonlinear amplification naturally coalesce. First, mechanical and electrical responses exhibited frequency selectivity. Independent experiments in which segments were exposed at varying positions along the cochlear spiral showed tuned responses that were consistent with the tonotopic map (43).

A key difference between the tuning curves measured in our preparation and those in the intact cochlea is the lack of asymmetry and a low-frequency flank. This distinction is an expected consequence of the absence of traveling waves (8, 57). In an intact cochlea, traveling waves introduce spatial coupling along the cochlear partition and result in characteristic asymmetries of the tuning curves. These emergent features stem from the unidirectional nature of wave propagation and constructive interference from upstream regions (58, 59). In our ex vivo system, traveling waves are absent and the dynamics reflect only interactions within a segment of sensory tissue.

The relationship of response magnitude to stimulus size was strikingly nonlinear over a range of weak to moderate stimulation. The response usually scaled logarithmically as the one-third power of the stimulus, with few or no examples of lower slope. In an active system operating near a Hopf bifurcation, that scaling should be obtained only at and around the bifurcation, whereas stimulation at frequencies other than the CF could yield any slope up to unity. Indeed, by using several different pure frequencies or by applying multiple stimulus frequencies simultaneously, we could demonstrate a power-law slope at the CF but a greater slope away from that frequency. As observed in earlier recordings with less sophisticated apparatus (44, 60), we encountered examples of power-law slopes greater than one-third. These higher slopes grew more prominent late in an experiment after extensive stimulation of the hair cells, a behavior similar to the progressive linearization of cochlear responses observed in vivo (51–53). Most notably, poorly dissected preparations that were visibly damaged only produced linear responses (*SI Appendix*, Fig. S8).

These behaviors contrast with that expected from saturating nonlinearities—such as the sigmoidal activation curve of mechanotransduction channels driving OHC electromotility—which do not impose a fixed power-law relationship. Furthermore, a saturating nonlinearity does not guarantee any minimum slope in the log–log plot of response magnitude versus stimulus intensity, in contrast with what we observed in healthy cochlear segments.

The third manifestation of the active process lay in the combination tones or distortion products evoked by the simultaneous presentation of two primary frequencies near the CF. As expected for a cubic nonlinearity, such stimuli evoked combination tones of forms 2·*f*_1_ − *f*_2_ and 2·*f*_2_ − *f*_1_. The remarkable feature of these responses was their scaling with the stimulus level: The combination tones grew in an approximately fixed ratio to the primaries. This behavior is characteristic of a dynamical nonlinearity—such as that associated with the active process of hair-cell transduction (46)—as a result of operation near a Hopf bifurcation. Although the cubic shape of the hair cell’s displacement–response relationship also leads to a cubic nonlinearity (61), that stationary nonlinearity grows superlinearly with respect to the primary tones as the stimulation level rises.

Two critical questions remain to be resolved: First, what are the exact roles of hair-bundle motility and membrane-based electromotility in effecting amplification and keeping the sensory tissue near a Hopf bifurcation? And second, how does the local active process integrate with traveling waves in the intact cochlea? The fact that a Hopf bifurcation appears both in individual hair bundles and in the mammalian cochlea as a whole does not exclude the possibility that other mechanisms have evolved to leverage criticality in mammals. Indeed, many types of dynamical systems can exist in the proximity of a Hopf bifurcation, and cochlear models that exclude hair-bundle motility can do so as well (62, 63). Our observation that regions containing OHCs exhibit mostly linear responses (Fig. 6 and *SI Appendix*, Fig. S12), whereas the RL and basilar membrane display compressive nonlinearity at low intensity levels suggests that additional mechanisms—possibly involving hair-bundle activity—contribute to the active process. Indeed, forms of nonlinear response persist in vivo when electromotility is impaired (64). Our data support a model in which amplification arises not as an emergent property of cochlea-scale organization, but as a local dynamical feature of the sensory epithelium itself.

Finally, our findings demonstrate that the mammalian cochlea harnesses the principle of criticality to enhance its response to stimuli. The result adds to the growing body of evidence that biological systems in general exploit this condition to optimize their functions (27–30). This idea aligns with what has been observed in nonmammalian vertebrates, whose hair cells exhibit active amplification through hair-bundle motility by operating in proximity to a Hopf bifurcation (2, 5–7), and even in insects, whose antennal hearing organs similarly exploit critical dynamics (65). The recurrence of these mechanisms across phylogenetically distant species suggests a conserved evolutionary strategy to the problem of detecting and encoding signals with high sensitivity and selectivity—one in which criticality serves as a universal biophysical principle for sensory processing (31).

By providing a high degree of experimental control and accessibility, our ex vivo preparation permits precise mechanical and electrical measurements and thus bridges the gap between cellular biophysics and systems-level behavior. In addition to offering an avenue to the identification of cellular mechanisms that regulate cochlear function, this work sets the stage for future exploration of the cellular and molecular intricacies of how the ear transforms mechanical signals into perception.

## Methods Summary

This paper provides a thorough description of the methods needed to create an experimental environment that accurately simulates the physiological conditions of the intact cochlea ex vivo, and therefore, contains detailed methodological information (*SI Appendix*, Extended Materials and Methods**). To assist readers who might not find these matters engaging, we will now present a summary of the methods.

### Experimental Preparation.

All experiments were conducted on three- or four-wk-old Mongolian gerbils (*Meriones unguiculatus*, Charles River Laboratories) under protocols approved by Rockefeller University’s Institutional Animal Care and Use Committee.

Each animal was killed by intraperitoneal injection of 200 mg·kg^−1^ pentobarbital sodium and phenytoin sodium (Euthasol^®^). After decoupling from the middle ear, each cochlea was promptly excised and placed into an oxygenated dissecting solution containing 155 mM Na^+^, 3 mM K^+^, 250 µM Ca^2+^, 1 mM Mg^2+^, 154 mM Cl^−^,1 mM HPO_4_^2−^, 5 mM HEPES, 3 mM pyruvate, and 10 mM D(+)-glucose, with a pH of 7.3 to 7.4 and an osmotic strength of approximately 310 mOsmol·kg^−1^.

Using a fragment of a razor blade 0.1 mm in thickness (Fine Science Tools), we separated the apical two turns by transecting the cochlea across its principal axis between the basal and middle turns. The upper turns were secured with isobutyl 2-cyanoacrylate adhesive (BOC Sciences) across a 1.5 to 2.0 mm-diameter hole in a plastic coverslip. We gained optical access to the cochlear partition by carefully removing the thin bone layers forming the middle turn’s ceiling and floor. To prevent leakage along the cochlear spiral and thus to confine electrical responses to the exposed cochlear segment, each cut end of the cochlear duct was sealed with cyanoacrylate adhesive. Finally, Reissner’s membrane was carefully removed (Fig. 1 *A* and *B* and *SI Appendix*, Fig. S1).

### Experimental Apparatus.

The ostensibly unperturbed segment of the sensory tissue, 500 to 1,000 µm in length, suspended between the osseous spiral lamina and the spiral ligament and secured to a rigid disc, was placed in a custom-made tripartite experimental chamber (Fig. 1*C* and *SI Appendix*, Fig. S2) The preparation was mounted vertically, such that the plane in which the disk and cochlear segment lay was perpendicular to the gravitational vector. This configuration prevented an uneven liquid load on the two sides of the preparation and therefore hydrostatic pressure that could displace the cochlear partition (42).

The experimental chamber comprised two compartments, one that adjoined the apical surface of the cochlear segment and one that abutted the basolateral surface. The outer compartment was exposed to atmospheric pressure, whereas the inner compartment was closed and housed the inputs from two speakers as well as the front of a microphone (*SI Appendix*, Fig. S2). Each fritted glass disc had a 2 mm-diameter central hole aligned with the preparation and the optical axis of a horizontal OCT system for measuring cochlear movements. A silicone-rubber gasket formed a tight seal between each compartment and the disc. Because of the hydrophilic nature of the fritted glass, each side of the preparation retained a thin layer of saline solution, and the reservoirs allowed periodic perfusion of liquid. The apical surface of the organ of Corti was immersed in oxygenated artificial endolymph containing 3 mM Na^+^, 155 mM K^+^, 26 µM Ca^2+^, 1 mM Mg^2+^, 153 mM Cl^−^, 1 mM HPO_4_^2−^, 5 mM HEPES, 3 mM pyruvate, and 10 mM D(+)-glucose, with a pH of 7.3 to 7.4 and osmolarity near 310 mOsmol·kg^−1^. The basolateral surface of the cochlear segment was exposed instead to oxygenated artificial perilymph consisting of dissecting solution with the CaCl_2_ concentration raised to 2 mM.

### Temperature Control.

The cochlear segment was maintained at the gerbil’s normal temperature (39) of 38 °C. Solutions were maintained at the same temperature with a water bath (181, Precision Scientific). After the experimental chamber had been assembled, the cochlear segment and surrounding fluids were heated with an infrared lamp. The temperature was measured before and after each stimulation protocol with a noncontact infrared thermometer (06-664-39, Traceable™ Noncontact Infrared Thermometer, Fisher Scientific).

### Stimulation and Electrical Recording.

Stimuli were synthesized by custom-written LabVIEW programs. After signal conditioning in a unity-gain power amplifier (SA1, Tucker-Davis Technologies), acoustic stimuli were generated by two earphones (ER-3C, Etymotic Research) coupled to the innermost compartment of the experimental chamber. The sound pressure in the air cavity was measured throughout each experiment with a free-field microphone (4939-A-011, Bruël & Kjær).

To reconstitute the ear’s endocochlear potential, we used a battery-operated current source to maintain a constant voltage of 80 to 100 mV across the preparation. A 1 mm Ag/AgCl pellet electrode was placed at the edge of the central hole in each fritted-glass disk, in contact with the saline solution near the preparation (Fig. 1*C* and *SI Appendix*, Fig. S2). We applied a steady endocochlear potential between the electrodes in the endolymphatic and perilymphatic compartments, with the former ordinarily positive relative to the latter.

A second pair of electrodes, also attached to the fritted-glass disks, measured the endocochlear potential and acquired the cochlear-microphonic potential excited by acoustic stimulation. The signal was preamplified X100 by a DC differential amplifier with low-pass filtering at 10 kHz (3000, A-M Systems), then amplified X200 and low-pass filtered at 5 kHz with an eight-pole Bessel filter (BM8.07, Kemo) before analog-to-digital conversion (PCIe-6353, National Instruments).

### Analysis of Cochlear Microphonic Responses.

Cochlear microphonics were elicited by delivering single‐ or multitone stimuli—tapered at onset and offset—to ensure smooth temporal profiles, either in 200 repeated presentations or as a single extended stimulus (for OCT compatibility). For each trial, the recorded time trace was converted to the frequency domain by means of a discrete FFT, and the magnitude and phase at the stimulus frequency were extracted. A Rayleigh test (*P* < 0.001) was then used to confirm that the phase distribution was nonuniform, thereby verifying phase‐locking of the response. Data points that did not meet this criterion were excluded (*SI Appendix*, Fig. S11). Detailed processing parameters are provided in *SI Appendix*, Extended Materials and Methods**.

### Optical Coherence Tomography.

To evaluate the condition of each preparation and measure its mechanical response to sound, we conducted OCT with a standard imaging system (GAN621, Thorlabs) whose optical path was horizontal. Equipped with a scanning objective lens (LSM02-BB, Thorlabs), the imaging head was mounted on a rigid rail facing the external fritted glass-containing compartment of the experimental chamber. An achromatic doublet lens of focal length 45 mm (AC245045B, Thorlabs) was placed 72 mm beyond the mounting plate of the scanning lens. A second achromatic doublet lens of focal length 19 mm (AC127019B, Thorlabs), spaced 169 mm from the mounting plane, confronted the specimen from a working distance of about 15 mm. The reference arm extended vertically, perpendicular to the imaging path. To compensate for the dispersion added by the doublet lenses, this arm included three 3 mm-thick plates of Schott NSF75 glass (ULWS062, United Lens Company; *SI Appendix*, Fig. S2). The measured axial and lateral resolution of our OCT system was 2.8 μm and 4.4 μm, respectively.]

Cross-sections (B-scans) of unstimulated preparations confirmed the integrity of the organ of Corti at multiple positions along an exposed cochlear segment (*SI Appendix*, Figs. S4 and S8). Sound-induced vibration profiles were acquired along a transect across the cochlear partition by measuring the movement for 1 s at each of 20 evenly spaced points (M-scans) along the chosen radius. A time trace was included in the dataset if it passed, with a *P*-value of 0.01 or less, a Rayleigh test designed to detect responses that were phase-locked to an acoustic stimulus. We tested the same pixels on the basis of the signal-to-noise ratio and found the Rayleigh test to be more reliable and rigorous.

## Supplementary Material

Appendix 01 (PDF)

## Data Availability

The datasets supporting the findings of this study have been deposited in Zenodo at https://doi.org/10.5281/zenodo.15747970 (66). All other data are included in the article and/or *SI Appendix*.
