# Supplementary material for: Amplification through local critical behavior in the mammalian cochlea
Source: Proc Natl Acad Sci U S A. 2025 Jul 14;122(29):e2503389122. doi: 10.1073/pnas.2503389122 (PMC12304976; doi:10.1073/pnas.2503389122)
Supplement: Supplementary file 1 — Appendix 01 (PDF) [file pnas.2503389122.sapp.pdf]

Supporting Information for:

Amplification through Local Critical Behavior in the Mammalian Cochlea

Rodrigo G. Alonso‡, Francesco Gianoli‡, Brian Fabella, & A. J. Hudspeth*

Howard Hughes Medical Institute and Laboratory of Sensory Neuroscience, The Rockefeller University, 1230 York Avenue, New York NY 10065 USA

*Corresponding author: Dr. A. J. Hudspeth (hudspaj@rockefeller.edu)

‡These authors contributed equally to the research.

This file contains:

- SI Extended Materials and Methods
- SI References
- SI Figs. S1 to S11

SI Extended Materials and methods

Requirements of an *in vitro* system for the study of the cochlear active process

To compare our *in vitro* results with those from the cochleae of living animals, we strove to provide an experimental environment that effectively mimicked the physiological environment of the intact cochlea. Moreover, we wished to conduct experiments on hair cells whose characteristic frequencies lay in the low kilohertz range that is critical for human verbal communication.

We earlier developed a simple version of the experiment chamber and successfully recorded cochlear microphonic potentials with a modest degree of compressive nonlinearity[1,2]. The system was subsequently analyzed and its characteristics were validated through finite-element analysis[3]. The preparation has also proven useful in physiological studies of various aspects of the mechanoelectrical-transduction process[4].

To accomplish the necessary objectives, an experimental system must meet an extensive set of requirements:

- The experimental animal must be readily obtainable at any time, at a reasonable price, and without an impact on natural populations. We have previously identified the Mongolian gerbil as suitable on these grounds[1,2].
- To facilitate acoustical stimulation, electrical recording, and microscopic observation, the isolated segment of the cochlea needs to bear hair cells with characteristic frequencies in the range of 1-10 kHz. The tonotopic map of the gerbil has been characterized exceptionally well[5,6], so it is possible to target hair cells of particular characteristic frequencies through anatomical landmarks during dissection.
- The cochlea must be sufficiently accessible that a functional segment can be dissected, mounted, and prepared for experimentation while the active process remains intact. In practice, this mandates a procedure that can be accomplished in about half an hour[1]. Productive experiments can ordinarily be conducted for at least an hour thereafter.

- The isolated cochlear segment must be rigidly secured so that its bony components do not vibrate at modest levels of stimulation and only the distensible cochlear partition moves significantly. Fixing the bony margins of a cochlear segment to a rigid plastic disk with cyanoacrylate adhesive meets this standard[1].
- The cochlear segment must separate two liquid-filled compartments and restrict ionic fluxes and electrical currents. A well-mounted segment of the gerbil's middle cochlear turn permits negligible leakage of liquids and displays a transepithelial resistance[1] of about 5 kΩ.
- One compartment of the experimental chamber must be filled with an artificial endolymph solution, the other with synthetic perilymph.
- The temperature is required to be maintained near 38 ˚C, the physiological level[7].
- A steady-state voltage has to be applied between the two compartments to simulate the normal endocochlear potential of 80-100 mV characteristic of young animals[8,9].
- The system must permit controlled stimulation with acoustic pressure. This requirement is critical for characterizing the active process, which can manifest itself only by generating mechanical forces of a magnitude comparable to those applied by the stimulation system. In other words, a mechanically active cochlear segment must be able to "push back" against the force applied by acoustic pressure. In engineering terms, the impedances of the stimulus system and cochlear segment must be approximately matched. The same consideration applies precisely in studies of the active motility of individual hair bundles, which can react mechanically against a flexible glass fiber[10], jet of liquid[11], or flux of photons[12], but not against a rigid stimulus probe.
- The electrical responses of hair cells must be readily accessible, for example, through recordings of cochlear microphonic potentials[13].
- The preparation needs to be optically accessible for light microscopy, especially with high-speed video cameras[2], for conventional interferometry, and for optical coherence tomography (OCT), which provides superior resolution of tiny, rapid movements in the cochlear partition.

General considerations

With the approval of Rockefeller University's Institutional Animal Care and Use Committee, we conducted experiments on three- or four-week-old Mongolian gerbils or jirds (*Meriones unguiculatus*, Charles River Laboratories) of both sexes. At three weeks of age, the cranial bone surrounding the cochlea was relatively soft and had not synostosed with that of the cochlear spiral. During the subsequent week, however, the bone grew harder and more brittle, so it became progressively more likely that dissection would lead to a fracture of the middle cochlear turn that ended the experiment. The age and sex of the animals were significant: males were somewhat larger than females at those ages, and their bones hardened sooner.

Tonotopic map of the gerbil's cochlea

The gerbil's cochlea represents frequencies[5] from about 0.3 kHz to 45 kHz, a range broadly comparable to that of humans and significantly below those of mice and rats. As assessed by the injection of individual cochlear nerve fibers, the tonotopic map displays a nearly exponential relationship of characteristic frequency to position along the three turns of the cochlear spiral[5]. A three-dimensional reconstruction of the cochlea by micro-computerized tomography[6] disclosed that the lateral surface of the middle turn encompasses characteristic frequencies from about 1 kHz to 4 kHz, an attractive range for mechanical and electrophysiological recording.

Dissection of a cochlear segment

Isolating a functional cochlear segment from a structure as complex and delicate as the cochlea is not easy. Nevertheless, the dissection procedure delineated below (*SI Appendix,* Fig. S1) usually results in preparation with satisfactory cochlear-microphonic responses and a reasonable appearance upon imaging by optical coherence tomography.

Initial preparations

- Euthanize an animal, remove the skin of its head, dislocate the mandible by horizontal cuts through the temporomandibular joints, reflect the posterior cranial musculature,

sever the external auditory meatus, and bisect the skull and brain sagittally with scissor cuts both dorsally and ventrally. Transect the skull immediately posterior to the eyes.

- Remove the brain, noting the internal auditory meatus—the entry point of cranial nerve VIII—adjacent to the ridge along which the tentorium inserts. The cochlear base lies immediately lateral to the internal auditory meatus; the helicotrema lies behind the ventral tip of the tentorial ridge.
- Periodically moisten each temporal bone with oxygenated dissection solution comprising 155 mM Na^+, 3 mM K^+, 250 µM Ca^{2+}, 1 mM Mg^{2+}, 154 mM Cl^-, 1 mM phosphate, 3 mM pyruvate, 10 mM D-glucose, and 5 mM HEPES at pH 7.35 and an osmolarity of 310 $mOsmol \cdot kg^{-1}$.

Isolating the two upper cochlear turns

- Although the procedures can be performed with a #11 scalpel blade, the best results are obtained with a sharply pointed fragment of a thin razor blade secured in a holder (14134-G, World Precision Instruments).
- Transect the modiolus basal to the middle turn by carefully inserting the blade into the internal auditory meatus orthogonal to the cochlear axis and carefully moving its tip back-and-forth. It is important not to insert the blade too far, for it can fracture the cochlear bone near the oval window.
- Using coarse dissection forceps, open the bulla by chipping away its bony wall until the cochlea is exposed.
- Use the scalpel blade's tip, or preferably a thin razor-blade fragment in a holder, to open a small hole in the bone overlying the helicotrema at the cochlear apex. This step may reduce pressure transients during the subsequent steps.
- Remove the flange of parietal bone forming the bulla's anterior wall at its junction with the bony outer wall of the cochlea.
- If desired, use a marking pen to draw an axial vertical line on the cochlea between the round window and oval window. This mark roughly demarcates the 2.5 kHz place in the middle turn and thus guides the opening of that turn's bony boundaries.

- Hold the cochlea with the apex uppermost by grasping the hard spicule of bone that protrudes axially below the round window. Use the scalpel blade or razor-blade fragment to shave bone from the medial surface of the cochlea, the flattened surface originally attached to the inner wall of the bulla. Likewise shave away the thicker bone adjacent to the oval window.
- Beginning at the oval window, use the tip of the blade to score the lateral bony wall of the cochlea basal to the junction between the middle and basal turns. Progressively deepen the incision with repeated application of the blade. The pigmentation of the stria vascularis provides a landmark for the approximate position of the cut.
- Notch the prominent bony flange in line with the score marks.
- Use the blade to score—and finally cut—the remainder of the thick ridge of temporal bone flanking the internal auditory meatus, freeing the top two turns of the cochlea from the rest of the specimen. This is the critical step in the entire dissection procedure: if it is successful, the remaining operations are relatively easy to accomplish. If the fracture of the bone extends apically into the middle turn or involves the bony roof of the basal turn, however, the preparation is unlikely to be useful (*SI Appendix,* Fig. S1A).
- Immerse the cochlear fragment with the two upper turns in dissection medium (*SI Appendix,* Fig. S1B).
- Trim any bony protrusions to leave the basal surface of the middle turn relatively flat and to facilitate placement into an experimental disk.

Securing the specimen to a plastic disk

- Insert the specimen, apex downward, into a 1.5-2.0 mm-diameter hole centered in a 12.5 mm-diameter, circular plastic disk cut from a plastic coverslip (12-547, Fisher Scientific). Orient the specimen so that the cochlear segment to be exposed lies at a suitable angle for microscopic imaging.
- After wicking away the excess solution surrounding the specimen with an absorbent wipe, use a mouth pipette (A5177, Sigma-Aldrich) to apply cyanoacrylate adhesive

from a broken microelectrode around the junction between the bone and the disk. Control the amount of adhesive carefully to prevent excessive spread, especially onto the exposed upper surface of the basal cochlear turn. Polymerize the adhesive by reapplying dissecting solution, then clear away any excess adhesive with forceps.

Exposing the basal aspect of the middle-turn cochlear partition

- Secure the disk in a petri dish filled with dissection solution and, if possible, transilluminate it with a darkfield condenser.
- Using moderately fine forceps, chip away the bony ceiling of the basal turn to expose the cochlear partition over one-tenth to one-quarter of the middle turn. For recordings near the 2.5 kHz place, which represents the approximate midpoint along the length of the organ of Corti, the appropriate window lies opposite the flattened bony wall of the cochlea and midway between the oval and round windows, about one-third turn apical to the severed orifice of the basal turn. Centered roughly at the vertical pen mark, the site is usually marked by a tiny radial artery on the bony apical surface of the bottom cochlear turn.
- Expel cyanoacrylate adhesive from a broken micropipette to seal the openings of the scala tympani at both ends of the exposed segment (see *SI Appendix, Extended Materials and Methods: Restriction of leakage across the cochlear segment*). In addition, seal both the scala vestibuli and the scala tympani at the position where the basal cochlear turn was amputated. Cover the exposed nerve fibers and the remainder of the exposed basal modiolar surface with adhesive, leaving a window for only the cochlear segment of interest (*SI Appendix,* Fig. S1C).

Exposing the apical aspect of the middle-turn cochlear partition

- Invert the disk and preparation in the petri dish to expose the cochlear apex.
- Expose the helicotrema by chipping away the delicate bone with fine forceps. Continue removing the lateral body wall of the apical turn, leaving the cochlear duct and prominent stria vascularis intact.

- Sever the duct and stria at the most basal exposed position, approximately opposite the vertical pen mark, and remove the soft tissue.
- Carefully chip away the lateral bony wall of the apical cochlear turn apical to the junction with the bony floor of that turn.
- Using fine forceps, perforate the thin bony floor of the apical turn and remove the delicate bone coextensive with the cochlear segment defined by the earlier dissection from the basal surface of the middle turn (*SI Appendix,* Fig. S1D).
- Tear open Reissner's membrane with a sharp micropipette to allow electrical access and a diffusional pathway for artificial endolymph.

Restriction of leakage across the cochlear segment

In order to prevent mixing of the solutions in the two compartments of the experimental chamber and to maintain the standing potential between them, it is important to seal the bone of the dissected cochlear segment tightly to the plastic mounting disk and to minimize leakage across the dissected specimen. This may be accomplished by careful application of a cyanoacrylate adhesive such as *n*-butyl 2-cyanoacrylate (Vetbond, 3M, St. Paul, MN; Histoacryl, B. Braun, Melsungen, Germany), 2-octyl cyanoacrylate (Dermabond, Ethicon Inc., Raritan, NJ), or a mixture of the two (PeriAcryl 90HV, GluStitch Inc., Delta, BA, Canada). Because we earlier found superior results with Iso-Dent[1], which is no longer commercially available, we prefer to use custom-synthesized isobutyl cyanoacrylate (BOC Sciences, Shirley, NY) colored with 5 mg/mL Sudan II dye and immediately centrifuged to remove hardened contaminants. This formulation also lacks the hydroquinone and other components found in some commercial products.

A pair of pipettes effective for the application of adhesive may be made by heating a glass capillary made for microelectrode fabrication (1B120F-3, World Precision Instruments, Sarasota, FL) in a small flame, then rapidly hand-pulling it to a total length of about 400 mm. Breaking the pipettes to a taper length of roughly 100 mm apiece results in internal tip

diameters of 100-150 μm, which is appropriate for injecting adhesive into the scalae exposed during dissection.

To slow the hardening of the cyanoacrylate, it is convenient to break a few millimeters from the end of each adhesive-filled micropipette immediately before use and to briefly dip the tip into 150 mM sodium acetate buffer solution at pH 5.2. The modest acidity inhibits polymerization until the adhesive is expelled, and the weakly acidic solution at a physiological osmotic strength apparently does not damage the hair cells. Repeated breakage allows a single pipette to be used for the application of adhesive at several sites.

Mounting the isolated cochlear segment

A key component of the experimental system is an experimental chamber consisting of a stack of three plastic segments. Each segment measures 48 mm in width and 40 mm in height. The second and third segments are identical, each with a thickness of 7 mm, whereas the first segment is distinct and has a thickness of 10 mm. Each of the three has a centered, cylindrical through hole 21 mm in diameter. The segments are produced by three-dimensional printing of high-temperature-resistant stereolithographic material (Accura® SL 5530, 3D Systems Corporation, Rock Hill, SC) (*SI Appendix,* Fig. S2B). Computer-assisted-design plans for the chamber are available on request.

The first segment of the chamber is mounted to a metal block by four 30 mm-long M3 bolts that also serve to clamp the chamber's segments together. The second and third segments bear four through holes at their corners that align them upon bolts protruding from the first segment. Two 2 mm-diameter stainless-steel tubes (89935K224, McMaster-Carr, Robbinsville, NJ), each 18 mm in length, are glued vertically into the upper surface of that segment to afford connections to speakers for acoustic stimulation. A 7 mm-diameter hole, 14 mm in length, on one side allows the insertion of a microphone to monitor the sound-pressure level at the cochlear partition.

To minimize the volume of liquid through which acoustic stimuli must travel and which must oscillate in response to stimulation, we capture a few hundred nanoliters of physiological

solution in the central bore of each of a pair of quartz frits (QPD20-4, Technical Glass Products), one apiece in the second and third segments of the chamber. Made by sintering finely crushed glass, a frit 20 mm in diameter and 3-5 mm in thickness readily imbibes ~ 300 μL of liquid, or ~30 % of its total volume. We grind a 3-4 mm-diameter hole through each frit's center, then secure the frit around its circumference with epoxy adhesive so that one surface is flush with that of the relevant segment of the experimental chamber and can fit snugly against the plastic disk clamped between the two frits. The opposite surface is thinned around the central hole so that its thickness is only 1 mm or so at the orifice where the cochlear segment is secured. The reservoirs of the second and third segments each hold about 1 mL of the appropriate solution and thus maintain appropriate concentrations of ions and other constituents through diffusion while thinning the layer of liquid overlying the specimen and allowing only a few hundred nanoliters to move freely.

Prior to mounting the preparation in the experimental chamber, we saturate the glass frit in the second segment of the recording chamber with the oxygenated physiological solution appropriate for the planned experiment, usually artificial endolymph. We center atop the frit a gasket of silicone elastomer (86435K123, McMaster-Carr, Robbinsville, NJ) roughly 30 mm in diameter and with a central hole about 4 mm in diameter, then position the specimen so that its apical surface protrudes through the hole and into the liquid-filled central bore of the frit. After positioning another gasket atop the specimen, we add the third segment of the chamber, ordinarily filled with perilymph, so that the basal surface of the specimen extends into the bore of that frit. Tightening nuts on the chamber's screws then clamps the plastic disk firmly between the two gaskets, which prevents electrical and ionic leakage between the two compartments of the experimental chamber. After the assembled chamber has been secured to a micromanipulator with the frits oriented vertically, we fill reservoirs in the second and third segments with the appropriate oxygenated physiological solutions. The micromanipulator is placed on a metal rail, electrodes are attached to impose the endocochlear potential and to monitor the microphonic potential, and tubes are connected to couple the stimulation speakers to the chamber's ports.

Provision of physiologically appropriate ionic solutions

Our artificial perilymph contains 155 mM Na^+, 3 mM K^+, 2 mM Ca^{2+}, 1 mM Mg^{2+}, 154 mM $Cl^–$, 1 mM phosphate, 3 mM pyruvate, 10 mM D-glucose, and 5 mM HEPES at pH 7.35 and with an osmolarity of 310 mOsmol·kg^{-1}. This solution is identical to the dissection solution save for the elevated Ca^{2+} concentration.

The artificial endolymph solution contains 3 mM Na^+, 155 mM K^+, 26 μM Ca^{2+}, 1 mM Mg^{2+}, 153 mM $Cl^–$, 1 mM phosphate, 3 mM pyruvate, 10 mM D-glucose, and 5 mM HEPES at pH 7.35 and with an osmolarity of 306 mOsmol·kg^{-1}. As a result of Ca^{2+} chelation by several forms of phosphate ion[14], the concentration of free Ca^{2+} is calculated to be near the physiologically appropriate value[15,16] of 20 μM.

Provision of an endocochlear potential

An endocochlear potential of 100 mV can be reconstituted by passing a current of approximately 20 μA between a pair of 1 mm-diameter silver-silver chloride pellet electrodes (EP1, World Precision Instruments). An electrode is placed at the inner edge of the moistened frit in each of the second and third segments of the experimental chamber. Because the electrode is less than 1 mm from a surface of the mounted cochlear partition, the voltage drop between the two is negligible.

Because the recording electrodes are directly exposed to the endocochlear potential, it is necessary to assert that potential with a low-noise system isolated from ground. We used a battery-operated circuit with two operational amplifiers. One is a unity-gain, non-inverting follower driven from the circuit's ground. The other, similar amplifier receives its input from the sweeper of a 10 kΩ variable resistor at the center of a voltage divider in which it is flanked by two 100 kΩ resistors. When the voltage divider is polarized by ±9 V, the system's output encompasses about ±430 mV.

Acoustic stimulation

Stimulus waveforms generated in a custom-written program (LabVIEW) are passed through a digital-to-analog converters (PCIe-6353, National Instruments Corporation, Austin, TX), a pair

of eight-pole Bessel filter (BM8.07, Kemo, Dartford, Kent, UK) with a low-pass cutoff of 10 kHz to remove extraneous frequency components, and calibrated active attenuators (SA1, Tucker-Davis Technologies, Alachua, FL) to two independent miniature speakers (ER3C, Etymotic Research, Lucid Audio, Ft. Worth, TX). The sound stimuli are then relayed through the tubing provided with the speakers to the inner compartment of the experimental chamber.

The system is calibrated by inserting into the same compartment a 1/4" microphone (4939-A-011, Brüel & Kjær). After amplifying the response (Nexus 2690-051, Brüel & Kjær) and recording the result as a function of frequency, we implement an inverse filter at the level of the computer to achieve a flat stimulus amplitude over the range 1-4 kHz.

Relationship of stimulus-pressure values to airborne sound pressure levels

In an intact mammal, sound is transmitted from the tympanum through the bones of the middle ear and to the oval window. The round window is meanwhile in contact with the middle-ear cavity, in which the mean pressure is near the atmospheric value. The pressure difference delivered to the oval window exceeds that at the tympanum by a factor of ~20 (26 dB)[17]. Moreover, owing to the properties of the cochlear traveling wave, the pressure close to the basilar membrane can be greater still, with a net gain of 100-1000 relative to the sound pressure at the ear canal [18].

In our preparation, which lacks the structures of the outer and middle ear and prevents the propagation of traveling waves, airborne pressure wave reaches the water interface that bathes the sensory epithelia. Due to the impedance mismatch between the two media, a small fraction of approximately 0.05% of the incident pressure is transmitted into the liquid It follows that the sound-pressure levels that we report represent stimuli that are smaller by at least 26 dB than the corresponding values under conventional recording conditions.

Recording of microphonic responses

The cochlear microphonic response, which predominantly reflects the activity of outer hair cells[19], is recorded with a second pair of pellet electrodes similar to that for asserting the endocochlear potential. These electrodes at the inner edges of the frits are only a fraction of a millimeter from the exposed cochlear segment. Signals are measured with a high-input-impedance amplifier (model 3000, A-M Systems, Sequim, WA) with a gain of 100X and a passband from DC to 5 kHz. For antialiasing purposes, an eight-pole sharp elliptic filter (BM8.13, Kemo) with a low-pass cutoff of 5 kHz and gain of 200X is imposed before analog-to-digital conversion at intervals of 10 μs.

The amplifier's direct coupling is required to simultaneously measure the constant endocochlear potential, which may be adjusted with the variable resistor. In addition, the signal provides an index of the condition of the experimental preparation. Outer hair cells ordinarily operate near a transduction-channel open probability of one-half[20,21,22], so a significant deviation from a symmetrical microphonic response waveform implies a problem such as an inappropriate Ca^{2+} concentration in the endolymph or a steady-state pressure difference across the cochlear segment.

Optical coherence tomography

To assess the health of each preparation and to measure the mechanical responses to acoustic stimulation, we perform optical coherence tomography (OCT) with a conventional imaging system (GAN621, Thorlabs, Lübeck, Germany) that is adapted for use with our optical system (*SI Appendix,* Fig. S2A). We ordinarily mount the experimental preparation with its basal surface and the surrounding perilymph in the outer compartment of the experimental chamber so that the basolateral aspect of the cochlear segment is directed toward the imaging optics. The apical surface is directed into the inner compartment of the experimental chamber, where it encounters endolymph. A second doublet lens 25.4 mm in diameter and of focal length 45 mm (AC245045B, Thorlabs) is held in an adjustable mount such that the nearest surface of the lens is about 100 mm beyond that of the first lens. The manufacturer's 18 mm-focal-length

scan lens (LSM02BB, Thorlabs), which is affixed to the scan head, is then situated with its front element 47.5 mm beyond that of the second lens. With this arrangement the microscope produces a real image on the camera of the OCT system. The magnification of the system is calibrated with the manufacturer's software and a specimen bearing three perforations separated by 800 μm (Thorlabs). The axial resolution of 2.8 μm was measured from the line width of an OCT A-scan reflected off a mirror surface. The lateral resolution was estimated using a resolution target (R2L2S1P1, Thorlabs) where it was possible to distinguish down to 228 line pairs (lp/mm), which corresponds to a resolution of approximately 4.4 μm.

The reference arm of the OCT system stands vertically, perpendicularly to the imaging path, and terminates in a retroreflector about 280 mm above the scan head. To compensate for the optical dispersion produced by the three imaging lenses, we place in the reference pathway of the OCT system three 3 mm-thick plates of Schott N-SF75 glass (UL-WS-062, United Lens Company) as well as the compensator meant for the system's scan lens. This arrangement compensates for most of the dispersion, leaving the software of the OCT system with a typical adjustment value of 150-175 of the manufacturer's arbitrary units.

Verifying the absence of a traveling wave in the cochlear segment

The principal simplification afforded by our experimental system was the suppression of traveling waves. Of the 11.5 mm-long spiral of the gerbil's organ of Corti[6], we ordinarily exposed only 500 μm or so, or 4 % of the total. For a typical recording centered at the characteristic place for 1.5 kHz stimulation, the tonotopic map[5] implies that the mechanically and electrically accessible hair cells in the segment should have represented frequencies of approximately 1–3 kHz. Two physiological observations confirmed this inference.

First, when the preparation was stimulated with a multitone zwuis stimuli of constant amplitude, the microphonic potential characteristically displayed a well-defined peak of enhanced responsiveness in the expected frequency range (Fig. 2A). And second, when a microphone was tightly sealed to the outer segment of the experimental chamber adjacent to a formaldehyde-fixed experimental preparation during acoustic stimulation, the measured

sound pressure displayed a similar plateau of heightened magnitude (*SI Appendix,* Fig. S5). The latter finding implies that, over a restricted passband, the isolated segment exhibited an enhanced acoustic transmittance by comparison to the remainder of the cochlea.

When activated by a traveling wave in an intact cochlea, a segment of the size that we exposed should encompass only a few cycles of oscillation. Both high-speed video micrography[2] and theoretical analysis[3] suggest that an isolated cochlear segment displays only a modest phase difference from one end to the other. When we captured the motion of the organ of Corti by OCT observation at a series of transects spanning a cochlear segment, we indeed observed a phase range of roughly ± 1 rad or $\pm 57^{\circ}$ (*SI Appendix,* Fig. S4). We, therefore, consider that the cochlear segment essentially oscillated as a single unit, with little traveling-wave behavior; as a consequence, the microphonic potential likely emerged from the summation of the nearly synchronous electrical responses of all of the active hair cells in the isolated segment. Because the gerbil's cochlea encompasses approximately 1100 inner hair cells and 4600 outer cells[6], the linear densities of the two are respectively 96 mm^{-1} and 400 mm^{-1}; a 500 μm-long segment therefore contains about 50 inner and 200 outer hair cells.

Analysis pipeline for the cochlear microphonics

To measure the response of cochlear segments across a range of sound pressure levels we delivered pure-tone stimuli—tapered at onset and offset to avoid discontinuities—for 200 repetitions. Each resulting cochlear microphonic (CM) time trace (*SI Appendix,* Fig. S11A) was transformed into the frequency domain by applying a discrete Fast Fourier Transform (FFT). For each trial, we extracted the magnitude and phase at the stimulus frequency and subjected the phase distribution from all 200 measurements to a Rayleigh test to determine whether the CM response was phase-locked to the stimulus. The Rayleigh test is the calculation of a confidence level (p-value) for the hypothesis that a certain distribution of phases comes from a non-uniform distribution (not pure noise). If this p-value is low enough, it is safe to assume that the phases are not uniformly distributed and that the time trace contains a phase-locked response to the stimulus[23,24]. Any data that did not match this criterion ($p>0.001$) were excluded

from further analysis (*SI Appendix*, Fig. S11B,C). We repeated this procedure for each sound-pressure level, thus generating a set of CM magnitudes as a function of sound pressure levels that were reliably phase-locked to the stimulus. The time traces where then averaged and the magnitude of the resulting FFT measured at the stimulus frequency was plotted against the corresponding pressures in log-log coordinates—effectively producing what is termed as a "level function." Performing the FFT on individual time traces first and then averaging the magnitudes led to the same results.

In some experiments, in order to accommodate the requirements of the OCT measurement apparatus, we delivered a single stimulus of longer duration instead of averaging shorted repeated ones. In this case, the steady-state portion of the CM response was chopped into segments of at least 500 ms each. Each segment was then zero-padded to extend its duration to match the length of the entire original signal. Importantly, we ensured that each segment retained its original position within the time trace, meaning its temporal alignment relative to the stimulus was preserved. From each padded segments we extracted the magnitude and phase at the stimulus frequency and performed the Rayleigh test on these phases to assess the reliability of the cochlear microphonic response.

Analysis of the minimum slope as a function of frequency across multiple experiments

Across multiple experiments we delivered pure tone stimuli ranging from approximately 600 Hz to 3 kHz to cochlear segments that localized in the accessible section of the middle turn of the gerbil'a cochlea, which represents frequencies[6] of 1-4 kHz. To estimate the slope of the level functions across experiments we took the validated data on magnitude *versus* pressure and grouped consecutive points in overlapping triplets or quadruplets. Each group was then fit with a least-squares line in doubly logarithmic coordinates. Fig. S9 illustrates this slope calculation in the case of triplets of points for a cochlear segment stimulated at 1.8 kHz. We rejected measured slopes whose standard errors was greater than 0.15 to ensure high confidence in the linear fit. This fitting procedure was repeated across 78 experiments (48 gerbils) to yielding a distribution of slope values that spanned the relevant frequency range (*SI Appendix*,

Fig. S9B). Finally, we used a moving window 50 Hz in width to identify the smallest recorded slopes at each frequency interval, and fitted these minimal slopes with a straight line, yielding an overall mean lower-bound slope near 0.33 ± 0.10. The minimal slopes were detected in response to frequencies of 1–5 kHz, which differ slightly from the characteristic frequencies expected from the middle turn of the gerbil's cochlea[6] (1–3 kHz), but are consistent with the portion of the tonotopic axis accessible in our experiments.

Alignment of multiple responses across experiments

Because of differences among animals, variations in cochlear dimensions, and other factors, it is difficult to compare absolute response magnitudes across preparations. We therefore focused on those exhibiting any instance of sublinear slope (< 0.5) at the characteristic frequency (*SI Appendix,* Fig. S9). For each such preparation, we plotted the sensitivity (FFT magnitude/sound pressure) as a function of the sound-pressure level and used an unsupervised algorithm to identify an "inflection point" separating two regimes.

A typical sensitivity curve displays nonlinear regime at lower sound pressure levels which will follow a power law with a negative exponent. For a dynamical system near a Hopf bifurcation this exponent is -2/3. At higher sound pressure levels, the response is expected instead to grow linearly with the stimulus where the sensitivity does not grow with the increased sound pressure and the exponent of the power law is null. We define the "inflection point" as where the linear fit of the sub-linear region at lower sound pressure levels intersects the linear fit of the linear regime at higher levels.

To select this "inflection point" we perform an unsupervised search based on an optimization algorithm based on the assumption that there exists a data point along the sensitivity curves that marks the boundary between the nonlinear and the linear regime. The following constraints were enforced: the nonlinear slope must be negative, while the other slope is fixed at 0. An optimization routine (e.g., L-BFGS-B) was used to minimize the sum of squared errors (SSE), subject to bounds on the slope and continuity at the inflection point. This unsupervised procedure yields a candidate (P_{INFL}, S_{INFL}) for each sensitivity curve, near the

transition between negative and zero exponents. If the inflection point lay outside the valid range of the data or if the residual sum of squares (RSS) exceeded a threshold (indicating a poor fit), the dataset was either re-initialized or excluded.

Each curve is then translated in the log-log space so that the inflection points overlap. We defined a common horizontal range in the aligned axis and discretized it into a finite array of points. For each aligned dataset, we performed linear interpolation from it onto this shared grid. This yields a uniform set of interpolated values across all experiments which was then used to calculate an average sensitivity curve (plus an estimate of variability using the standard deviation) in the aligned log-log space.

To visualize how the exponent changes along the averaged curve, we used a sliding window of 4 consecutive data points and for each performed a linear fit (least-squares) to approximate local slopes of the average curve. The standard error of the slope in each window was estimated by standard linear regression formulas. A t-distribution was used to compute 95% confidence intervals. The half-width of the confidence interval was used to plot the error bars and “fill-between” bands around the slopes.

SI References

1. Chan, D. K. & Hudspeth, A. J. Ca2+ current-driven nonlinear amplification by the mammalian cochlea in vitro. *Nat. Neurosci.* **8**, 149–155 (2005).
2. Chan, D. K. & Hudspeth, A. J. Mechanical responses of the organ of corti to acoustic and electrical stimulation in vitro. *Biophys. J.* **89**, 4382–4395 (2005).
3. Nankali, A. & Grosh, K. Simulating the Chan-Hudspeth experiment on an active excised cochlear segment. *J Acoust Soc Am* **142**, 215 (2017).
4. Lin, W.-C., Macić, A., Becker, J. & Nam, J.-H. Asymmetric vibrations in the organ of Corti by outer hair cells measured from excised gerbil cochlea. *Commun Biol* **7**, 600 (2024).

5. Müller, M. The cochlear place-frequency map of the adult and developing Mongolian gerbil. *Hear Res* **94**, 148–156 (1996).

6. Hutson, K. A., Pulver, S. H., Ariel, P., Naso, C. & Fitzpatrick, D. C. Light sheet microscopy of the gerbil cochlea. *J Comp Neurol* **529**, 757–785 (2021).

7. Mele, J. A. Temperature Regulation and Bioenergetics of the Mongolian Gerbil Meriones unguiculatus. *American Midland Naturalist* **87**, 272 (1972).

8. Schmiedt, R. A. Effects of aging on potassium homeostasis and the endocochlear potential in the gerbil cochlea. *Hear Res* **102**, 125–132 (1996).

9. Suryadevara, A. C., Schulte, B. A., Schmiedt, R. A. & Slepecky, N. B. Auditory nerve fibers in young and quiet-aged gerbils: morphometric correlations with endocochlear potential. *Hear Res* **161**, 45–53 (2001).

10. Martin, P. & Hudspeth, A. J. Active hair-bundle movements can amplify a hair cell's response to oscillatory mechanical stimuli. *Proc. Natl. Acad. Sci. U.S.A.* **96**, 14306–14311 (1999).

11. Tobin, M., Chaiyasitdhi, A., Michel, V., Michalski, N. & Martin, P. Stiffness and tension gradients of the hair cell's tip-link complex in the mammalian cochlea. *Elife* **8**, (2019).

12. Abeytunge, S., Gianoli, F., Hudspeth, A. J. & Kozlov, A. S. Rapid mechanical stimulation of inner-ear hair cells by photonic pressure. *Elife* **10**, e65930 (2021).

13. Corey, D. P. & Hudspeth, A. J. Analysis of the microphonic potential of the bullfrog's sacculus. *J. Neurosci.* **3**, 942–961 (1983).

14. Recillas, S., Rodriguez-Lugo, V., Montero, M., Viquez-Cano, S. Hernandez, L., Castaño, V. M. Studies on the precipitation behavior of calcium phosphate solutions. *J. Ceramic Proc. Res.* **13**, 5–10 (2021).

15. Bosher, S. K. & Warren, R. L. Very low calcium content of cochlear endolymph, an extracellular fluid. *Nature* **273**, 377–378 (1978).

16. Ikeda, K., Kusakari, J., Takasaka, T. & Saito, Y. The Ca2+ activity of cochlear endolymph of the guinea pig and the effect of inhibitors. *Hear. Res.* **26**, 117–125 (1987).

17. de La Rochefoucauld, O., Kachroo, P. & Olson, E. S. Ossicular motion related to middle ear transmission delay in gerbil. *Hear Res* **270**, 158–172 (2010).

18. Dong, W. & Olson, E. S. Detection of Cochlear Amplification and Its Activation. *Biophysical Journal* **105**, 1067–1078 (2013).

19. Dallos, P. Response characteristics of mammalian cochlear hair cells. *J Neurosci* **5**, 1591–1608 (1985).

20. Kirk, D. L., Moleirinho, A. & Patuzzi, R. B. Microphonic and DPOAE measurements suggest a micromechanical mechanism for the 'bounce' phenomenon following low-frequency tones. *Hear. Res.* **112**, 69–86 (1997).

21. Patuzzi, R. & Rajan, R. Does electrical stimulation of the crossed olivo-cochlear bundle produce movement of the organ of Corti? *Hear. Res.* **45**, 15–32 (1990).

22. Bobbin, R. P. & Salt, A. N. ATP-gamma-S shifts the operating point of outer hair cell transduction towards scala tympani. *Hear. Res.* **205**, 35–43 (2005).

23. Versteegh, C. P. C. & Van Der Heijden, M. The Spatial Buildup of Compression and Suppression in the Mammalian Cochlea. *JARO* **14**, 523–545 (2013).

24. Cooper, N. P., Vavakou, A. & van der Heijden, M. Vibration hotspots reveal longitudinal funneling of sound-evoked motion in the mammalian cochlea. *Nat Commun* **9**, 3054 (2018).

SI Figures and figure legends

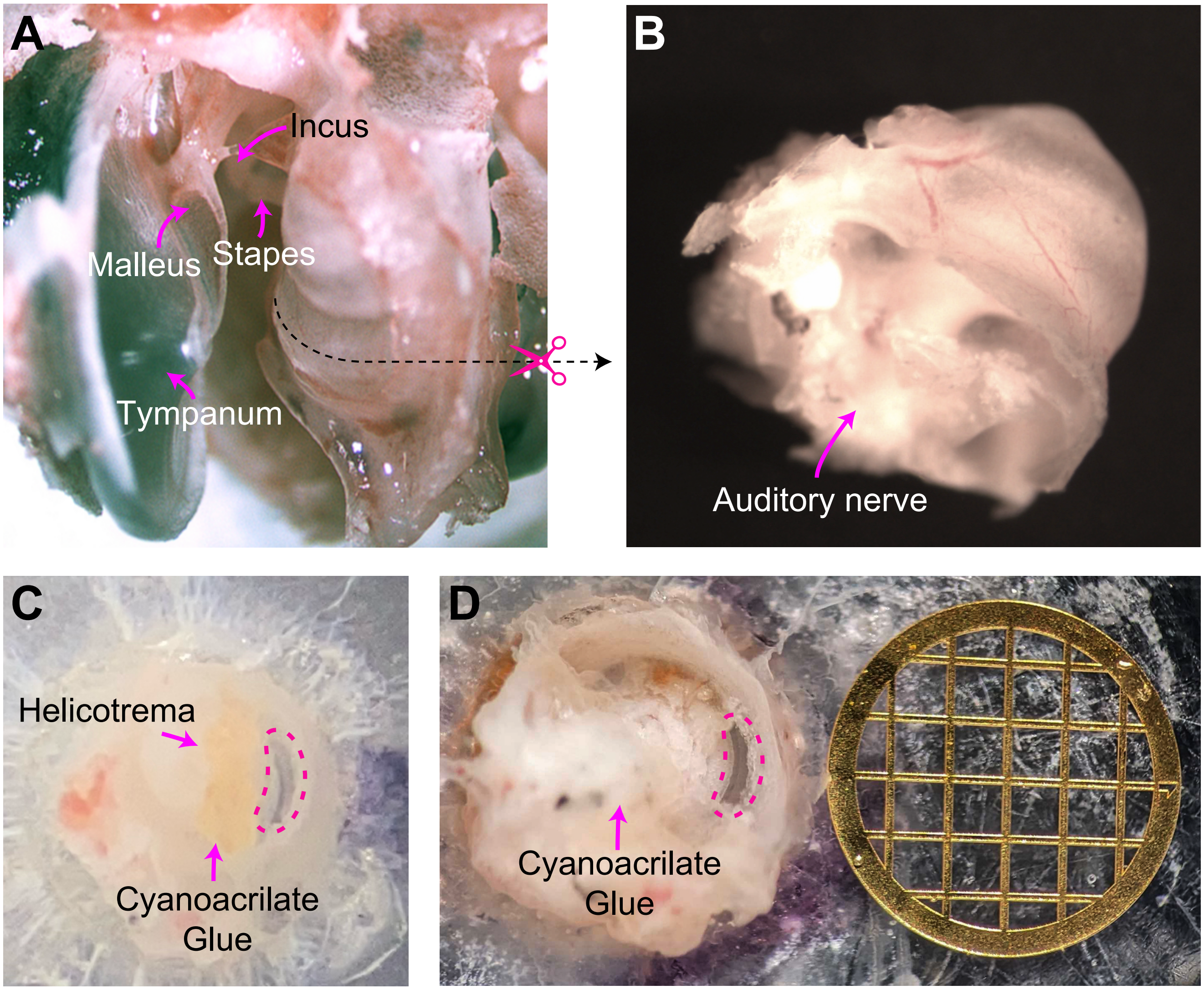

Fig. S1: The cochlear dissection and mounting protocol. A, Opening the bulla affords a view of the cochlea with the tympanum, malleus, incus, and stapes still attached. A dashed line marks the site of the first incision, which transects the cochlea to separate the basal and middle turns. **B,** After disconnection of the apical two turns of the cochlea, the auditory nerve is visible. **C,** In an apical view of the dissected cochlear partition, a "window" where the bony ceiling has been removed (dashed line) exposes the cochlear partition. The cyanoacrylate adhesive securing the specimen is visible in orange. **D,** A basal view shows the dissected cochlear

segment mounted on a plastic disk and secured with cyanoacrylate adhesive. A conventional electron microscopy grid 3 mm in diameter and with 500 μm windows calibrates the image.

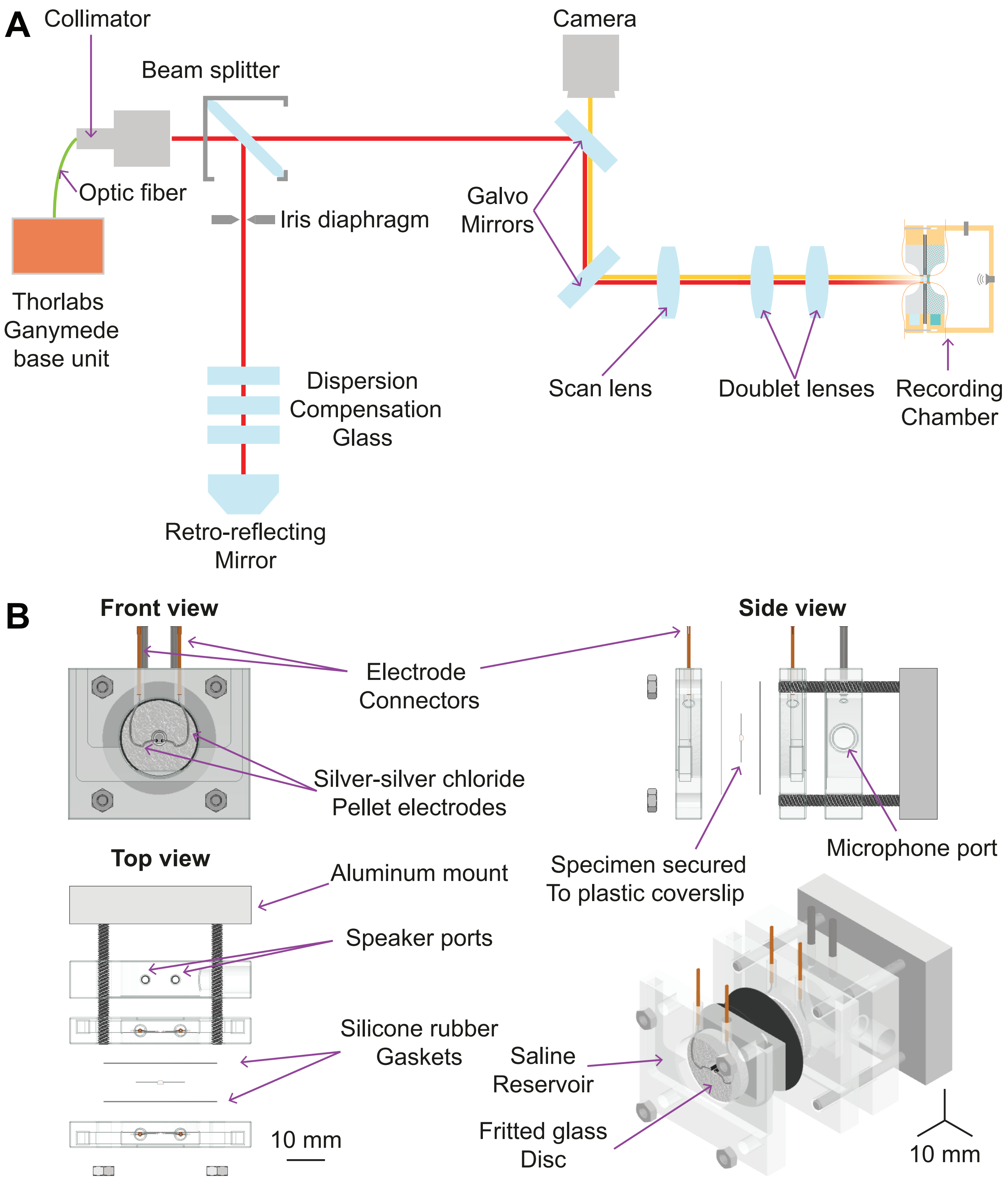

Fig. S2. Schematic diagram of the modified OCT imaging system and experimental chamber. A, The cochlear segment at the far right is mounted such that its basolateral surface, bathed in perilymph, faces the imaging optics in the outer compartment, whereas the apical

surface faces the endolymph-filled inner compartment. The optical path achieves extensive magnification with two lenses in addition to the system's standard scanning lens. The reference arm of the OCT system, which is oriented vertically, compensates most of the optical dispersion from the lenses with three glass plates and a glass disk. **B**, Computer-assisted design (CAD) schematics of the three-compartment experimental chamber are shown in front, top, side, and isometric views. Key components are labeled with purple arrows. The chamber's segments are stacked and clamped together with M3 bolts attached to an aluminum mount. Silicone rubber gaskets ensure that the compartments are tightly sealed. The second and third compartments are identical, while the first includes ports for speaker and microphone connections. The fritted glass disks, positioned in the second and third compartments, minimize the volume of free liquid in contact with the specimen and electrodes while maintaining ionic conditions through diffusion with larger reservoirs. The chamber was 3D-printed using high-temperature-resistant stereolithographic material, and its design supports precise and stable mounting of the cochlear segment for electrophysiological and vibrational measurements.

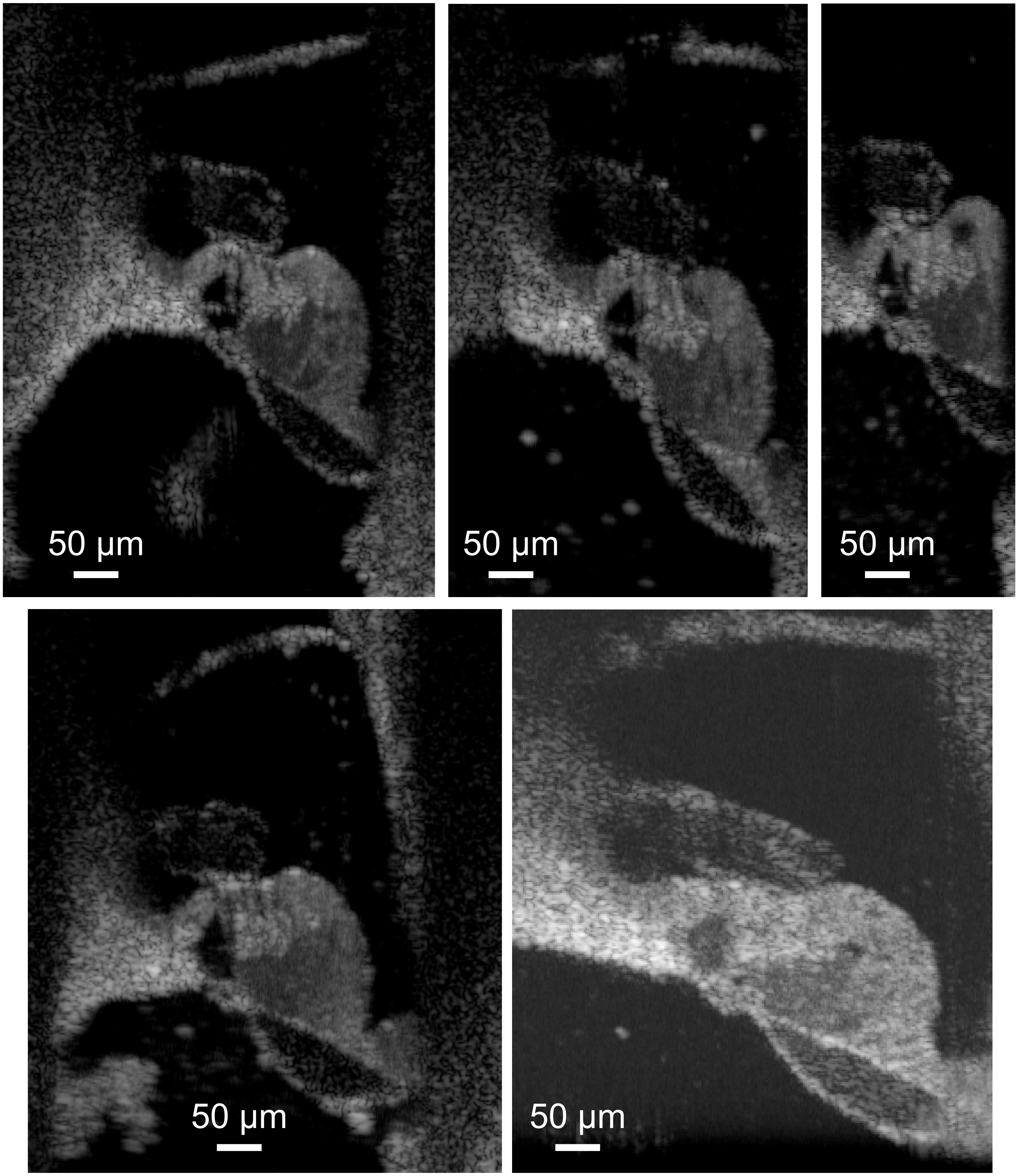

Fig. S3. Examples of healthy cochlear preparations. Reflectance B-scans from successful dissections display well-defined cellular and extracellular components with no signs of cellular damage. The tectorial membrane remains properly apposed to the reticular lamina and the

architecture of the organ of Corti appears intact. Such healthy preparations usually display the various manifestation of the active process.

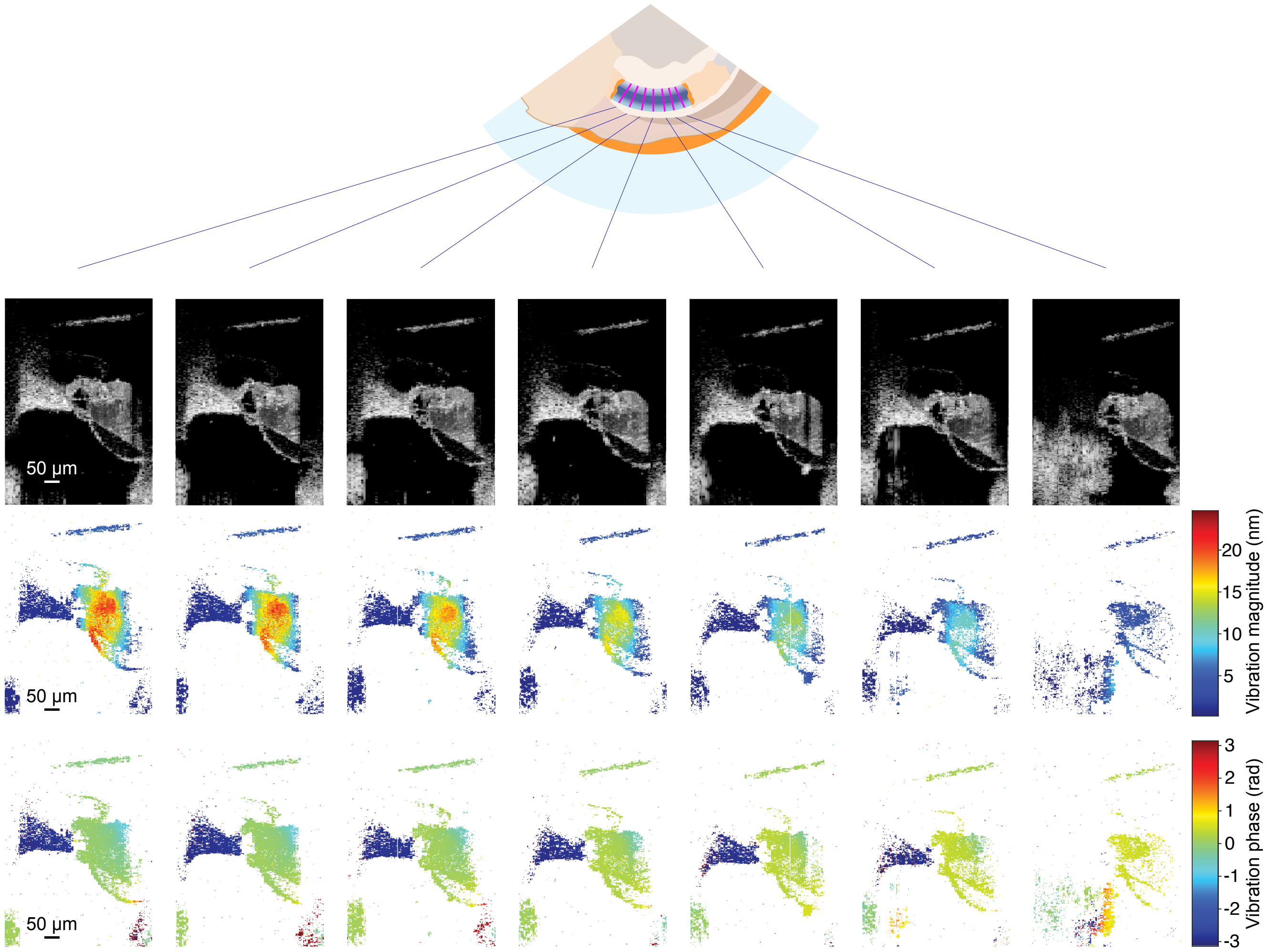

Fig. S4: Absence of a traveling wave. Seven transects (B scans) across a cochlear segment are displayed in columns, each comprising three images. The top row, the reflectance profile of the optical section through the organ of Corti, highlights key subcellular structures. The middle row displays the vibration magnitude at a stimulus frequency of 1520 Hz and intensity of 72 dB SPL. A prominent "hotspot" of large vibration is visible at the juncture between the outer hair cells and Deiters' cells. The bottom row, which depicts the phase profile in radians, documents consistent phase behavior across the organ of Corti. The data suggest that the cochlear segment oscillates as a unit with minimal evidence of a traveling wave.

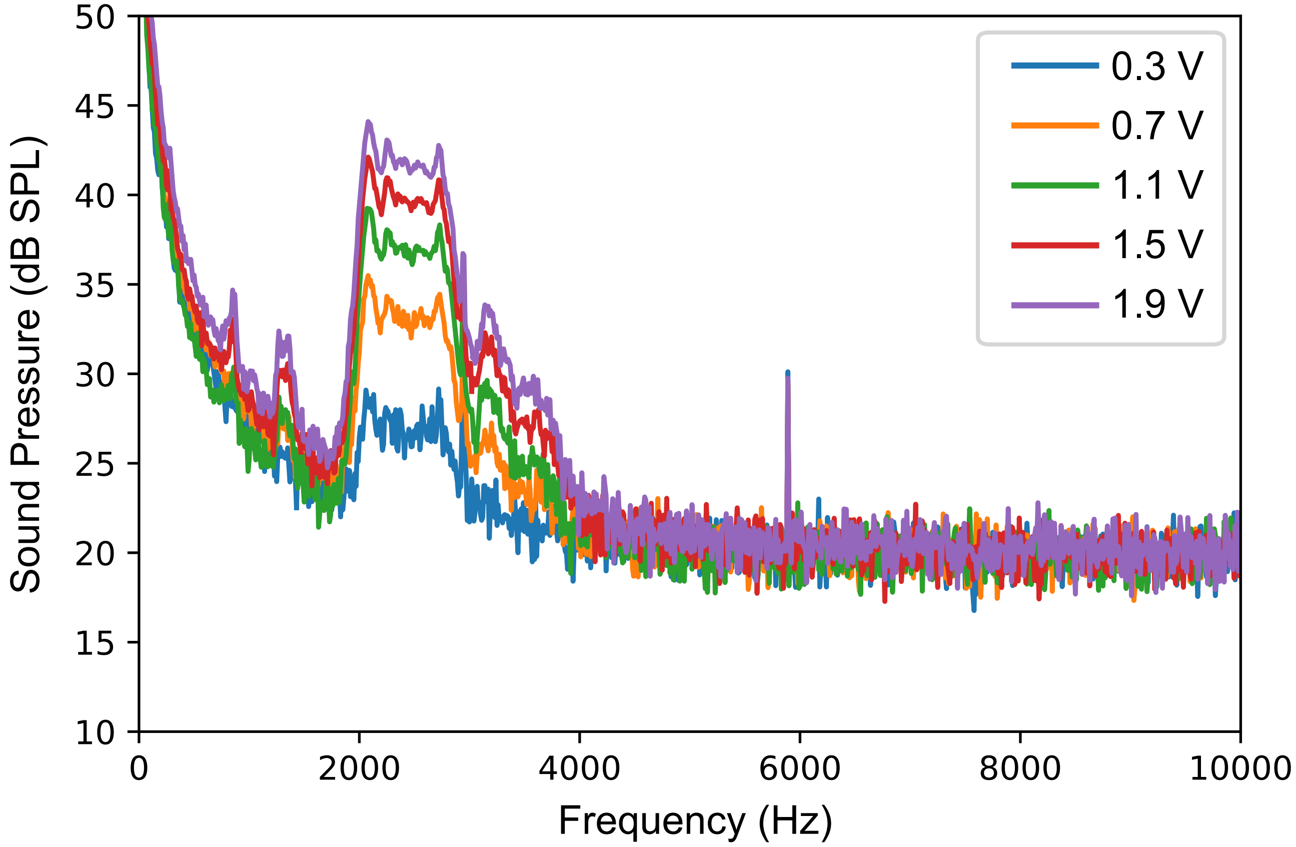

Fig. S5. Calibration of sound-pressure level in the experimental chamber. The plot shows the sound-pressure level as a function of stimulus frequency measured in the experimental chamber during acoustic stimulation. The calibration was performed with white-noise stimuli and a cochlear segment fixed in formaldehyde. The five curves correspond to speaker excitations ranging from 0.3 V to 1.9 V. A plateau of heightened magnitude over a restricted passband (2–3 kHz) demonstrates enhanced acoustic transmittance of the fixed cochlear segment, which is consistent with the expected frequency-tuned response of the exposed cochlear segment.

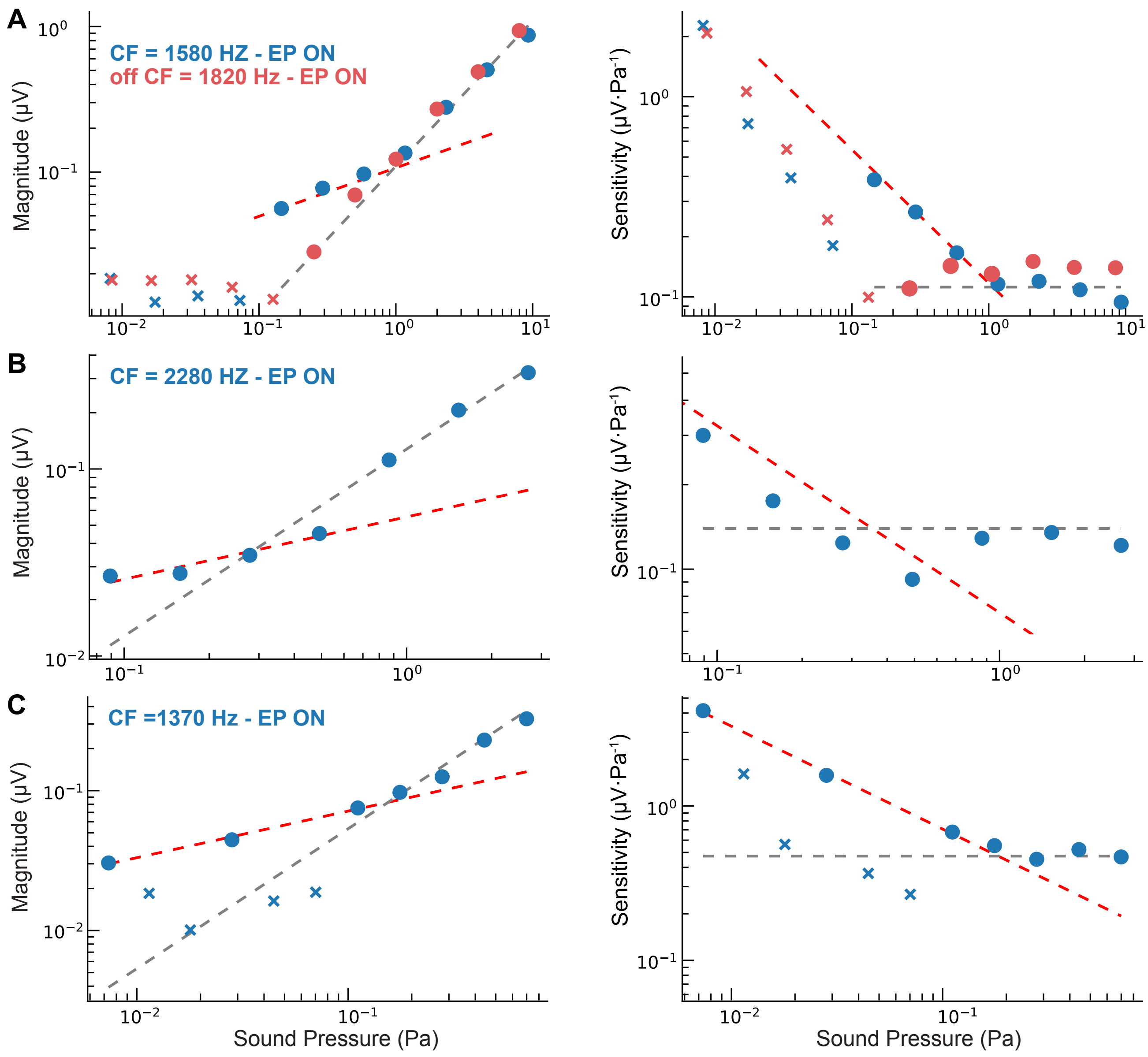

Fig. S6. Compressive behavior of cochlear microphonic responses. A, For a cochlear segment tuned to 1580 Hz, a doubly logarithmic plot (left) displays the magnitude of the microphonic response as a function of sound pressure. The blue points represent responses at the characteristic frequency with an endocochlear potential (EP) of 100 mV, whereas the red points depict responses to 1820 Hz. The right plot shows the preparation's sensitivity as a function of sound pressure with a corresponding color coding. At the characteristic frequency of 1580 Hz (blue), the response exhibits nonlinear behavior, whereas at a frequency of 1820 Hz (red), the response transitions to linear behavior. **B** and **C**, Data from two other preparations show a similar trends. The dots represent phase-locked responses that pass a Rayleigh test

($p < 0.001$), whereas crosses indicate responses that are statistically indistinguishable from noise ($p > 0.001$).

.

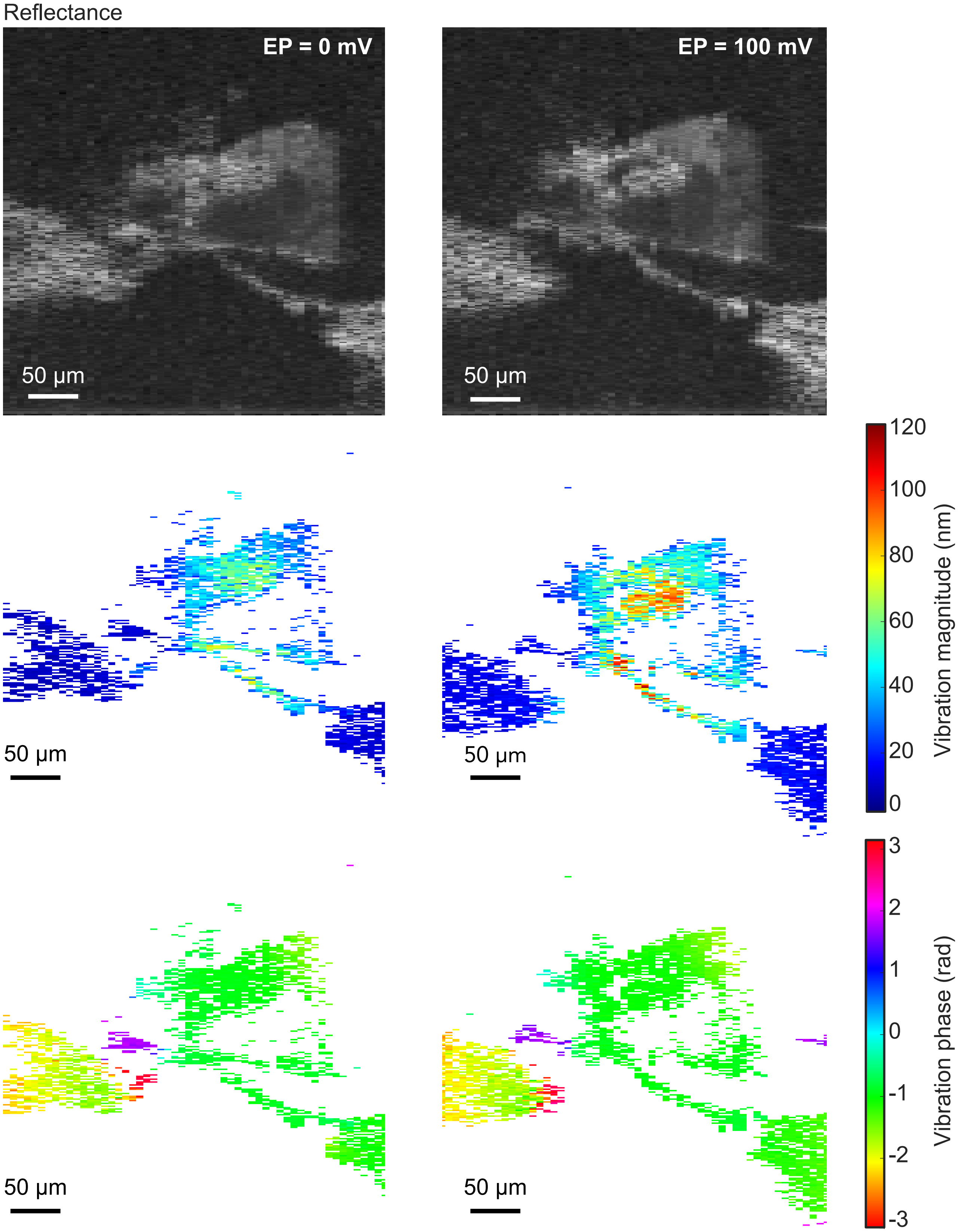
Reflectance
EP = 0 mV
EP = 100 mV
50 µm
50 µm
50 µm
50 µm
50 µm
50 µm
120
100
80
60
40
20
0
Vibration magnitude (nm)
3
2
1
0
-1
-2
-3
Vibration phase (rad)

Fig. S7: Intra organ vibration with and without the endocochlear potential. This Fig. illustrates the vibratory response of a cochlear segment to a 1.4 Pa stimulus at 1207 Hz. For the three images at the left, there was no endocochlear potential; the images to the right involved a potential of 100 mV. The top row shows reflectance B-scans for the sake of orientation. The middle row, which displays vibration magnitudes, demonstrates that the normal endocochlear potential significantly enhances the motion of the cochlear partition. A prominent hotspot of heightened vibratory magnitude signals mechanical activity. The bottom row, which represents the vibration phase, shows little sensitivity to the potential.

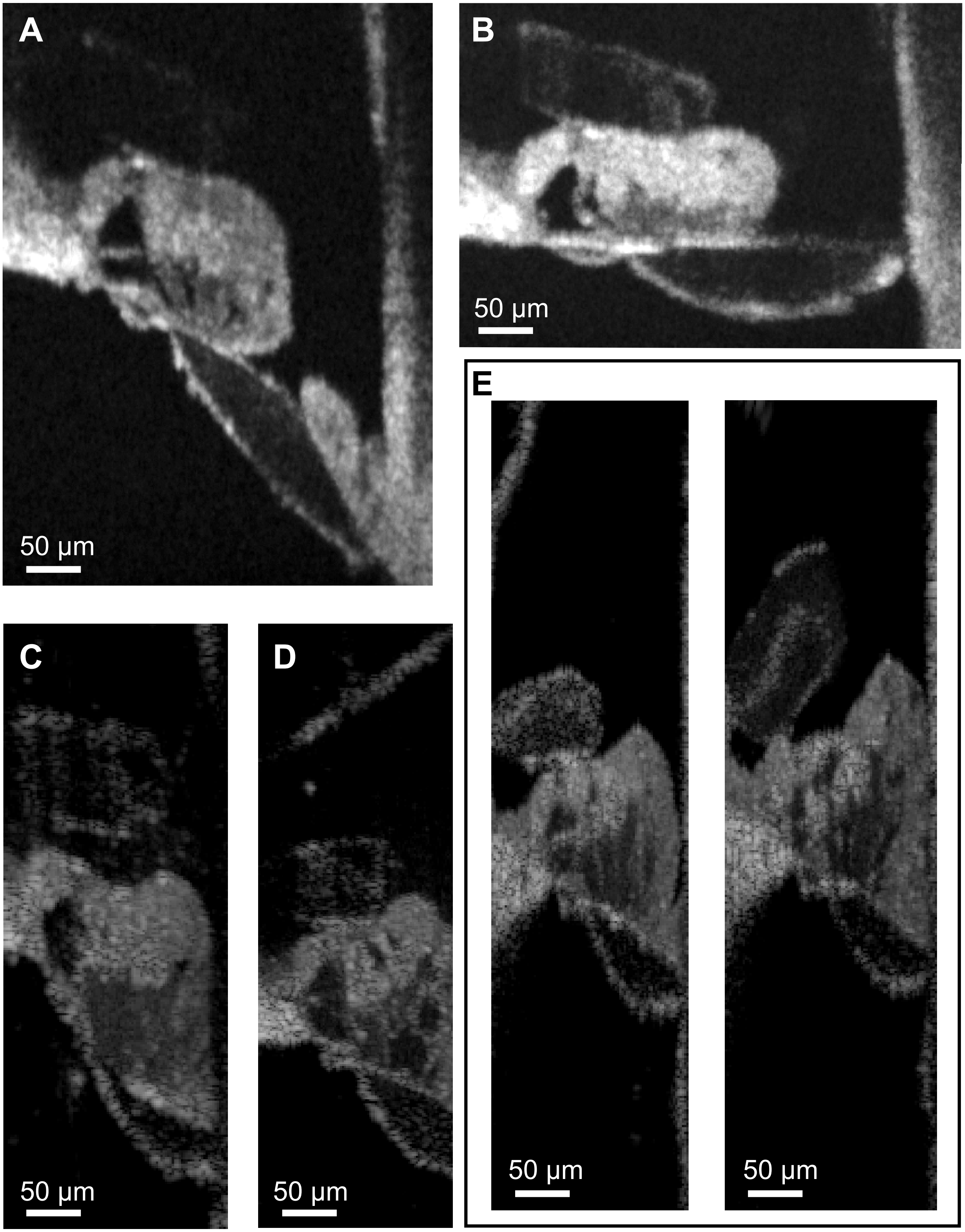
A
50 µm
B
50 µm
C
50 µm
D
50 µm
E
50 µm
50 µm

Fig. S8. Examples of unhealthy cochlear preparations. None of these preparations exhibited signs of active mechanics. **A and B,** Reflectance B-scans illustrate instances in which the cells of Claudius were accidentally torn away during dissection. **C,** In a preparation whose tectorial membrane was inadvertently displaced from the reticular lamina, the hair bundles were doubtlessly damaged. **D,** A B-scan shows dark "holes" that represent damage to Deiters' and Hensen's cells. **E,** A reflectance B-scan (left) shows a healthy preparation at the outset of an experiment. After prolonged stimulation, however, the same preparation (right) displays a displaced tectorial membrane and cellular deterioration within the organ of Corti.

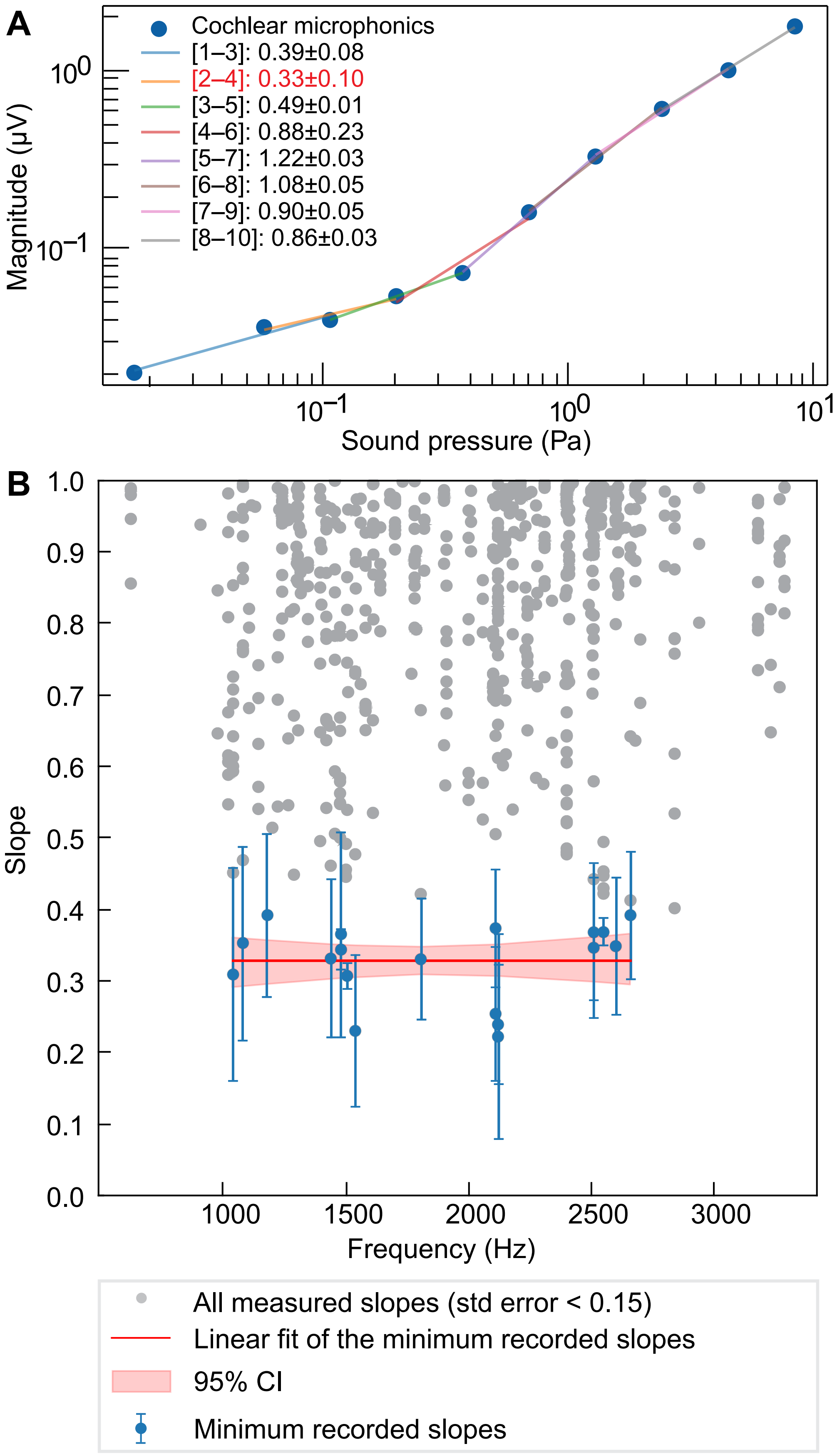

A
Cochlear microphonics
[1–3]: 0.39±0.08
[2–4]: 0.33±0.10
[3–5]: 0.49±0.01
[4–6]: 0.88±0.23
[5–7]: 1.22±0.03
[6–8]: 1.08±0.05
[7–9]: 0.90±0.05
[8–10]: 0.86±0.03
Magnitude (μV)
Sound pressure (Pa)
B
Slope
Frequency (Hz)
All measured slopes (std error < 0.15)
Linear fit of the minimum recorded slopes
95% CI
Minimum recorded slopes

Fig. S9: Minimum slopes measured across experiments. A, Example of a level function (Magnitude vs Sound pressure). In this case, the cochlear segment was stimulated with sine stimuli at 1800 Hz. The data were grouped in overlapping triplets of consecutive points (e.g. from the first to the third point [1–3]) and a linear fit is performed in loglog space (colored lines). The slope was determined using an ordinary least squares method and the standard error was determined from the covariance of the matrix. The minimum measured slope in this experiment was 0.33 ± 0.10 (red text). **B**, The measured slopes across 78 experiments (48 gerbils) as a function of the stimulus frequency. The frequencies explored went from approximately 600 Hz to 3300 Hz. All slopes were calculated using the approach described in panel a, using both triplets and quadruplets of consecutive and data points. All slopes whose standard error was larger than 0.15 were excluded from the analysis. A moving window of 500 Hz with a step-size of 10 Hz was used to select the smallest slopes recorded across experiments (blue dots with error bars). These smallest recorded values were encountered in a range of frequency from about 12.5 kHz. The linear fit on the slopes (red line, with 95% confidence intervals in pink) gives: fit y = mx +q, with m = 0.0 ± 0.0, and q = 0.32 ± 0.03. The mean slope across the minimum slopes recorded was 0.328 ± 0.009 with 95% confidence intervals of 0.31 and 0.35.

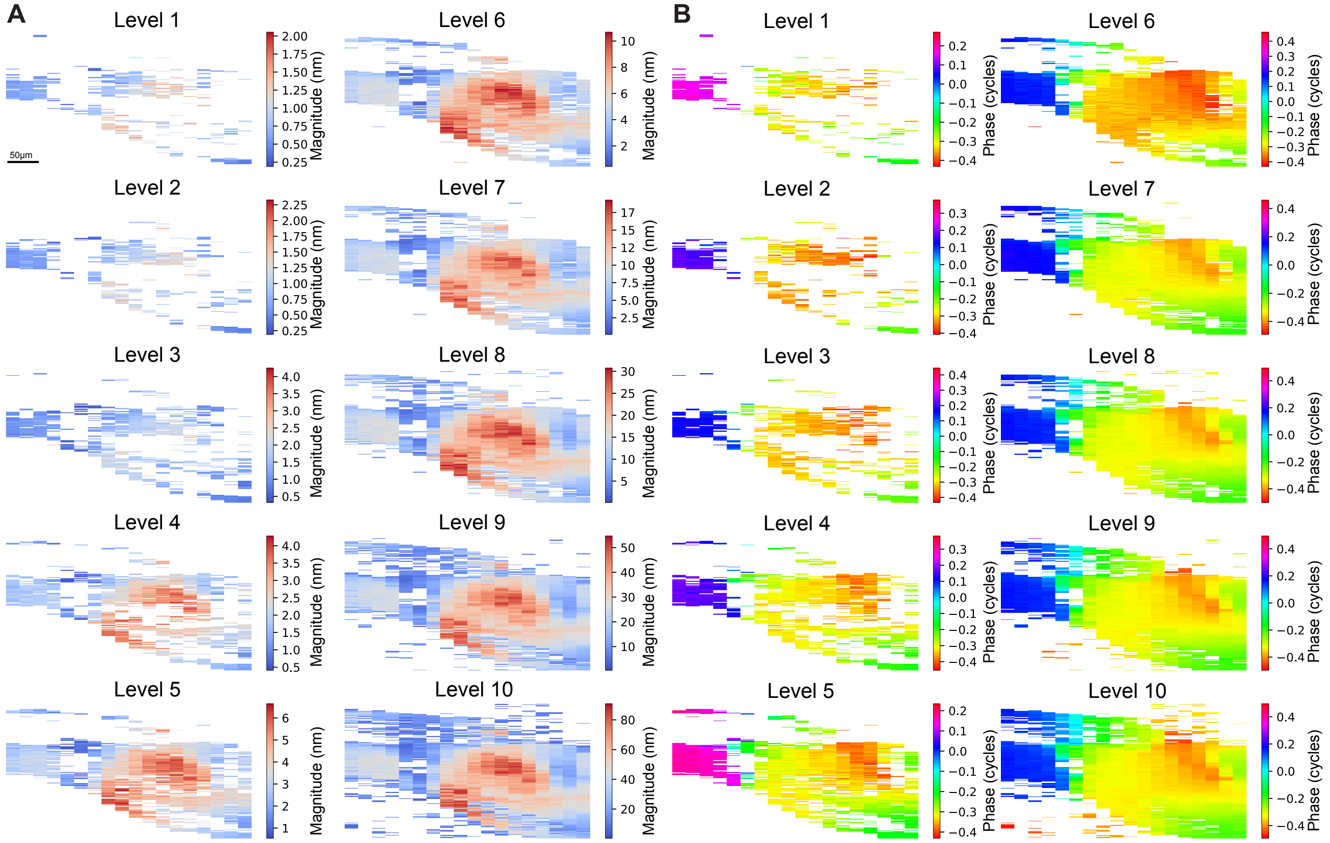

Fig. S10: Extended data from the preparation of Fig. 4. A, B-scans from the middle of an exposed cochlear segment display the vibratory magnitude at 20 equally spaced positions extending radially outward from the modiolus. Stimulation with pure tones at 2410 Hz increased exponentially from 0.08 Pa (level 1) to 4 Pa (level 10). Levels 1–5 are shown in the left column and levels 6–10 in the right column. **B,** Corresponding maps highlight the phase response of the organ of Corti at the characteristic frequency. These data provide a detailed view of the nonlinear vibratory behavior of the cochlear segment. This extended analysis offers greater details concerning the micromechanical complexity and level-dependent responses of the organ of Corti.

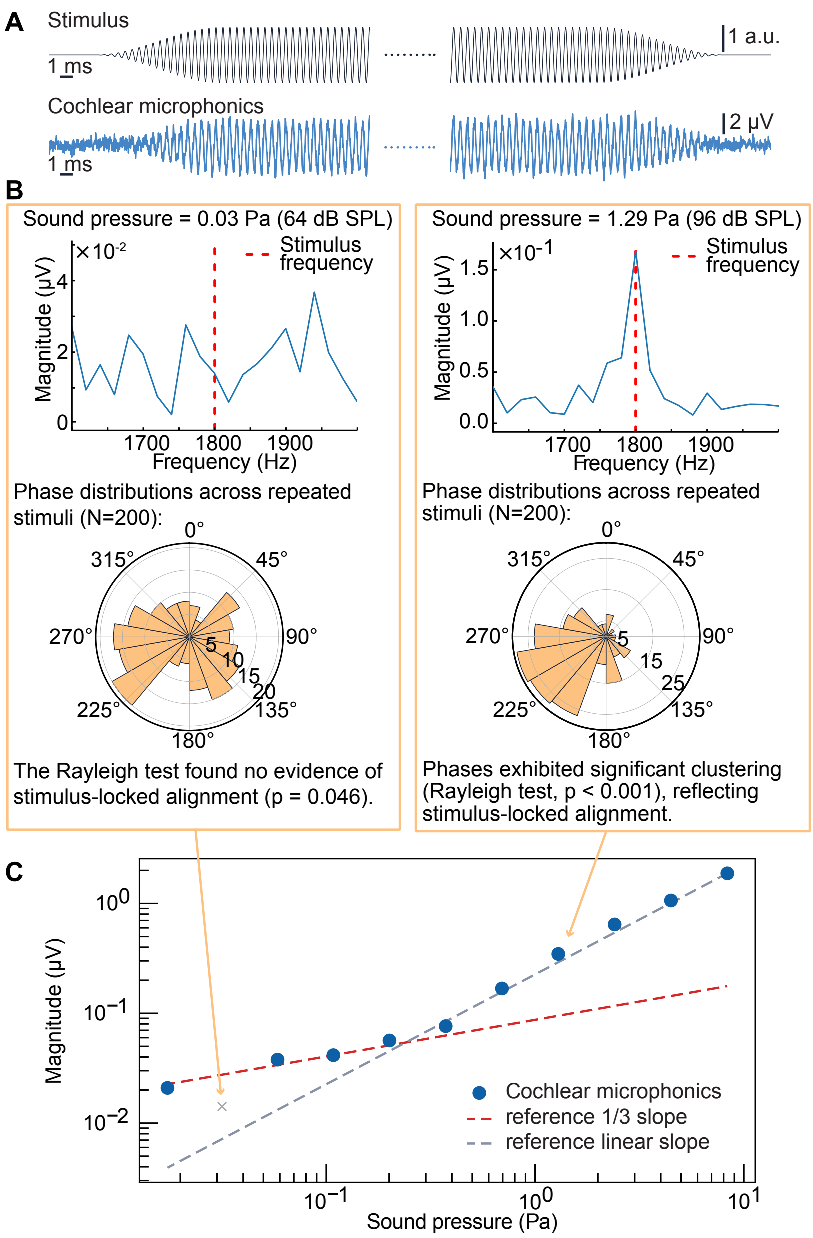

Fig. S11: Analysis of cochlear microphonic responses. A, Representative stimulus for a single tone stimulus at frequency 1800 Hz (top black trace) and the average cochlear microphonic response of 200 stimulus repetitions (bottom, blue trace). Stimuli are tapered at the onset and offset to avoid discontinuities. In this example, the sound pressure delivered was of approximately 8 Pa. **B**, Example of the FFT analysis for the cochlear microphonics responses to two sound pressure levels: 0.03 Pa (left box) and 1.29 Pa (right box). The magnitude and phase of the FFT was extracted for each of the N=200 repetitions. The distributions of the phases (orange polar plots) were used to determine whether the cochlear

microphonic response for each sound pressure level would be considered for the following steps of the analysis or rejected. To do so we applied the Rayleigh test on the phase distribution and rejected points whose p value was larger than 0.001. **C**, Example of a so-called level function (FFT magnitude vs Sound pressure). The second intensity level (gray cross), corresponding to a stimulus of 0.03 Pa did not pass the Rayleigh test and therefore it was rejected.

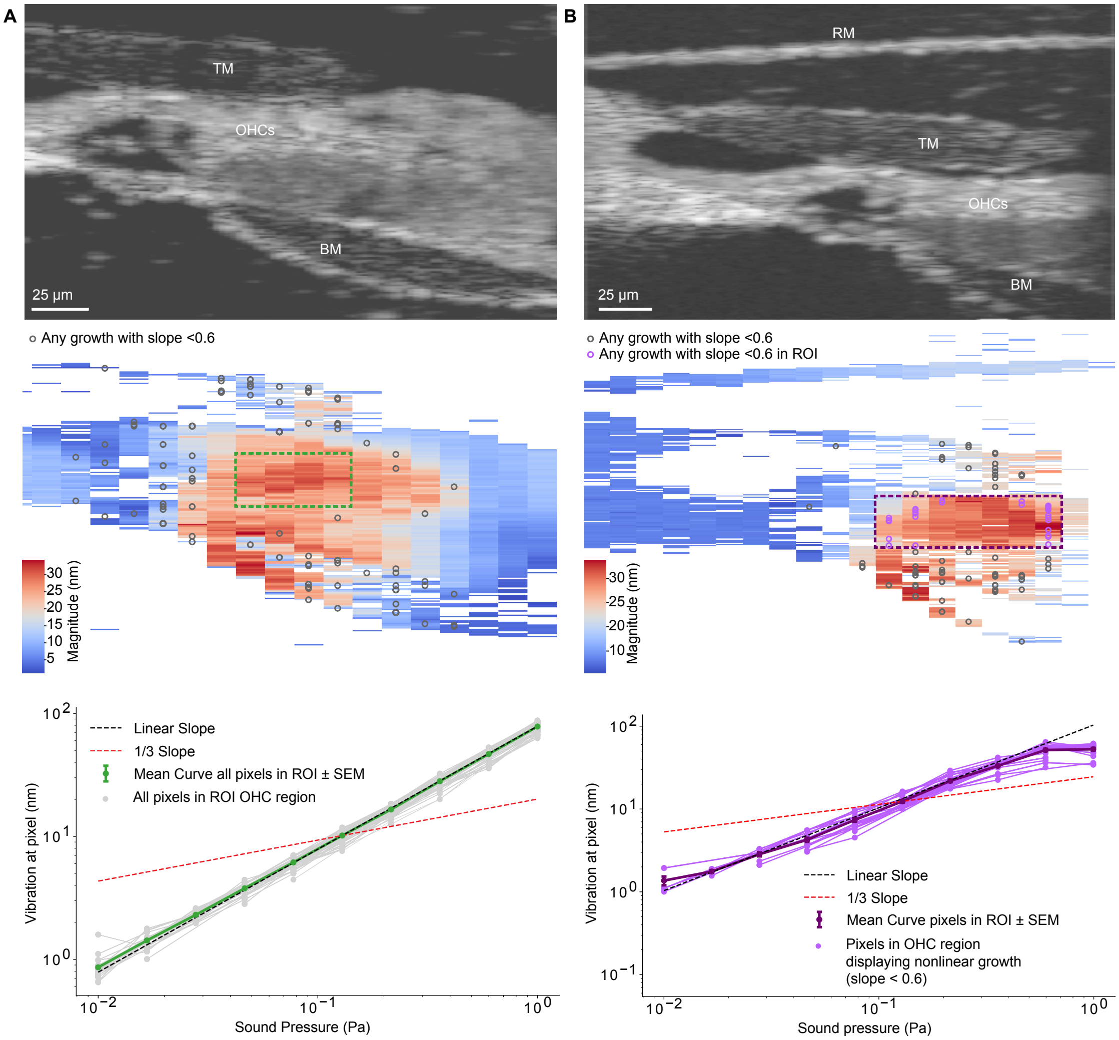

Fig. S12: Predominantly linear growth in the OHC region with instances of saturating responses. Top panels: Reflectance OCT images of the organ of Corti in two cochlear segments. The tectorial membrane (TM), basilar membrane (BM), and outer hair cells (OHCs) are labeled for orientation. The Reissner's membrane (RM) is labeled when visible. Middle panels: Vibration amplitude maps recorded at a stimulus level of 0.36 Pa, near the characteristic frequency of each segment (2510 Hz in A; 1231 Hz in B). In both segments, compressive nonlinear growth localizes primarily to the BM and reticular lamina, consistent with previous observations (see Fig. 6). In panel A, the region of interest (ROI) encompassing the OHCs (green dashed box) shows linear growth across stimulus levels. In panel B, although most nonlinear pixels again cluster in the BM and reticular lamina, a subset of pixels within the

OHC ROI (purple dashed box) exhibit signs of saturation, indicated by magenta circles marking pixels with sublinear growth (slope < 0.6). Bottom panels: Vibration magnitude as a function of sound pressure. In panel A, responses from all pixels within the OHC ROI are shown in gray, with the average (± SEM) overlaid. In panel B, only the pixels identified as nonlinear (with any measured local slope in log-log < 0.6) are plotted. Most OHC regions displayed linear growth (Fig.6 and panel A), but occasional instances of saturation were observed at higher stimulus levels (panel B).

Relative growth of cubic distortion products.

	Mean microphonic amplitude															
	Primaries (µV)					Cubic distortion products (µV)					Ratio (%)					Mean ratio (%)
	Level 1	Level 2	Level 3	Level 4	Level 5	Level 1	Level 2	Level 3	Level 4	Level 5	Level 1	Level 2	Level 3	Level 4	Level 5	All levels
Gerbil 1	2.10	2.54	2.98	3.41		0.11	0.13	0.17	0.24		5.30	5.12	5.64	7.07		5.78
Gerbil 2	0.14	0.29	0.33	0.35		0.05	0.06	0.06	0.08		32.53	19.69	17.69	24.23		23.53
Gerbil 3	0.67	0.88	1.13	1.39	1.71	0.07	0.07	0.18	0.23	0.30	10.69	8.46	15.58	16.62	17.43	13.75
Gerbil 4	1.03	0.93	0.74	1.44	1.47	0.07	0.07	0.06	0.14	0.13	7.14	7.16	8.18	9.86	8.64	8.19

**Total mean**	12.82
**SD**	7.89

Table S1. This table summarizes the mean microphonic amplitude (*µ*V) of responses to primary tones and nonlinear cubic distortion products across five sound pressure levels (1–5) of increasing magnitude in four independent gerbil cochlear preparations (Gerbils 1–4). For each preparation, the primary response corresponds to the mean amplitude elicited by two pure tones centered at the characteristic frequency. Distortion product amplitudes were quantified at first-order (cubic) frequencies. The third set of columns shows the percentage ratio of distortion product amplitude relative to the primary amplitude at each level. The rightmost column reports the average ratio across levels. The bottom right inset displays the overall mean and standard deviation.
